# Oxidative Stress-Related Mechanisms in Schizophrenia Pathogenesis and New Treatment Perspectives

**DOI:** 10.1155/2021/8881770

**Published:** 2021-01-23

**Authors:** Evgeny A. Ermakov, Elena M. Dmitrieva, Daria A. Parshukova, Daria V. Kazantseva, Alisa R. Vasilieva, Liudmila P. Smirnova

**Affiliations:** ^1^Laboratory of Repair Enzymes, Institute of Chemical Biology and Fundamental Medicine, Siberian Division of Russian Academy of Sciences, Novosibirsk 630090, Russia; ^2^Laboratory of Molecular Genetics and Biochemistry, Mental Health Research Institute, Tomsk National Research Medical Center of the Russian Academy of Sciences, Tomsk 634014, Russia; ^3^Siberian State Medical University, Tomsk 634055, Russia

## Abstract

Schizophrenia is recognized to be a highly heterogeneous disease at various levels, from genetics to clinical manifestations and treatment sensitivity. This heterogeneity is also reflected in the variety of oxidative stress-related mechanisms contributing to the phenotypic realization and manifestation of schizophrenia. At the molecular level, these mechanisms are supposed to include genetic causes that increase the susceptibility of individuals to oxidative stress and lead to gene expression dysregulation caused by abnormal regulation of redox-sensitive transcriptional factors, noncoding RNAs, and epigenetic mechanisms favored by environmental insults. These changes form the basis of the prooxidant state and lead to altered redox signaling related to glutathione deficiency and impaired expression and function of redox-sensitive transcriptional factors (Nrf2, NF-*κ*B, FoxO, etc.). At the cellular level, these changes lead to mitochondrial dysfunction and metabolic abnormalities that contribute to aberrant neuronal development, abnormal myelination, neurotransmitter anomalies, and dysfunction of parvalbumin-positive interneurons. Immune dysfunction also contributes to redox imbalance. At the whole-organism level, all these mechanisms ultimately contribute to the manifestation and development of schizophrenia. In this review, we consider oxidative stress-related mechanisms and new treatment perspectives associated with the correction of redox imbalance in schizophrenia. We suggest that not only antioxidants but also redox-regulated transcription factor-targeting drugs (including Nrf2 and FoxO activators or NF-*κ*B inhibitors) have great promise in schizophrenia. But it is necessary to develop the stratification criteria of schizophrenia patients based on oxidative stress-related markers for the administration of redox-correcting treatment.

## 1. Introduction

Schizophrenia is a complex and heterogeneous mental disorder. The heterogeneity of schizophrenia is associated with a wide range of causative biological pathways. However, the factor that unites these biological pathways is oxidative stress (OS). However, there is still no definite opinion on whether OS is the primary cause of the disease, or it occurs secondarily under the influence of environmental factors or long-term treatment. Nonetheless, it is generally accepted that OS plays an essential role in the pathogenesis of schizophrenia. In the first part of this comprehensive review, we will analyze the various mechanisms of schizophrenia pathogenesis associated with oxidative stress. In the second part, we will consider the effect of antipsychotic therapy on the parameters of redox balance, as well as review the prospects for the use of antioxidant therapy, and also propose new therapeutic strategies for redox correction based on transcription factor-targeting drugs.

## 2. Oxidative Stress in the Central Nervous System

Currently, there are a large number of facts that indicate the development of pronounced oxidative stress in various diseases of the central nervous system. It is due to a combination of many important factors and characteristics of the nervous tissue. The most significant of these is the high intensity of oxidative metabolism since 90% of the brain's energy needs are provided by aerobic processes [1]. Also important is the high content of unsaturated lipids in the nervous tissue [2] and metals of mixed valence (especially iron) [3], the participation of free radicals in neuroregulation [4], and the ability of several mediators and hormones to generate reactive oxygen species (ROS) [5].

The development of radical oxidative reactions in the nervous tissue is mainly local and depends on the metabolic characteristics of a particular type of tissue. CNS neurons are one of the primary consumers of glucose, oxygen, and ATP, high levels of which are necessary for maintaining membrane potentials, synthesizing neurotransmitters, and ensuring reorganization of synaptic connections and synaptic plasticity in postnatal development. All this provides an extreme sensitivity of neurons to OS; also, neurotransmitter metabolism itself generates prooxidants [6].

An imbalance of Ca^2+^ contributes to the aggravation of OS in neurons. ROS block the Ca^2+^ pumps of the endoplasmic reticulum and neurolemma, leading to an excessive concentration of Ca^2+^ ions in the cytoplasm of the neuron [7]. Intracellular Ca^2+^ regulates the release of neurotransmitters in the synaptic terminals, thus modulating synaptic activity and plasticity. In schizophrenia, there is a disruption of the synaptic transmission and plasticity [8], including the disruption of the N-methyl-D-aspartate receptor (NMDAR) activity, which is also modulated by Ca^2+^. Besides, Ca^2+^ ions activate nNOS and the formation of NO, CO_3_, and NO_2_ anions that trigger neurodegeneration processes, via the heat shock protein 90 and apoptosis activation [9].

Increasing the concentration of the intracellular Ca^2+^ ion activates phospholipase A_2_. Phospholipase A_2_ hydrolyzes membrane phospholipids, which are rich in cell membranes and myelin oligodendrocytes, by oxidizing polyunsaturated fatty acids (PUFAs) [10]. During phospholipase hydrolysis, PUFA is released from the membrane and further participates in signal transduction directly or after conversion to bioactive derivatives. PUFAs and their mediators regulate brain processes, such as neurotransmission, neurogenesis, neuroinflammation, and neuron protection. Besides, in neurons, PUFA is used as a substrate for the synthesis of ATP by *β*-oxidation due to the higher yield of ATP, compared to the oxidation of glucose and lactate [11].

Metals with mixed valence (especially iron and copper) can contribute to the development of OS in neurons [12]. Iron in brain tissues is necessary for metabolic processes, synthesis of aminergic neurotransmitters, and synaptic connections in neurons [13]. However, the high content of Fe^2+^ in the central nervous system is potentially toxic due to iron-generated ROS in an oxygen-rich environment. Also, metals with mixed-valence properties can bind directly to DNA, changing the properties of reparative proteins. Neurons contain a “labile” pool of Cu^+^(•), which is essential for transmitting cellular signals and excitability of neurons. Also, Cu^2+^ is a significant cofactor for enzymes. The high content of Cu^+^(•) ions in neurons (from 0.1 *μ*M to 1.3 *μ*M) encourages Cu^2+^-catalyzed protein oxidation and may be associated with a toxic increase in peroxidase activity, which can generate CO_3_ through HOOCO_2_ and thiol oxidase activity [14]. Copper ions can also participate in the generation of OH^•^ radicals by reacting with hydrogen hydroperoxide [15].

Having the factors mentioned above of vulnerability to OS, neurons have extremely weak antioxidant protection. Neurons have 50 times less catalase content compared to hepatocytes. The content of reduced glutathione (GSH) is ~50% lower in neurons compared to other cells (for example, ~5 *μ*M in neurons compared to 10-11 *μ*M in hepatocytes) [16]. The reduced ability to synthesize GSH due to the low content and activity of the transcription factor Nrf2 (nuclear factor erythroid 2-related factor 2), which binds the promoter, is responsible for the low level of cytosolic GSH in neurons. Cortical neurons have been shown to express Nrf2 approximately 100-1000 times less than astrocytes [17]. Additionally, the neuronal activity of Nrf2 is limited by the high content of the Cullin 3 protein, contributing to the proteasome degradation of Nrf2 [18]. All this contributes to the accumulation of ROS in neurons.

GSH enhances GABA-activated responses of inhibitory neurons via GABA receptors. Low GSH levels can lead to a decrease in GABA-mediated feedback inhibition and affect the normal function of GABAergic neurons in the prefrontal cortex by removing inhibitory effects on neurons in this area, which may be the cause of positive symptoms of schizophrenia [19]. In patients with schizophrenia, a decrease in the level of glutathione in the prefrontal cortex has been shown [20].

Astrocytes, in contrast, play a significant role in providing antioxidant support to neighboring neurons, and redox regulation of the Nrf2 astrocyte pathway is a potent homeostatic regulator of a large cohort of Nrf2-regulated antioxidant genes that are expressed by these cells. Even in an inactive state, astrocytes actively express antioxidant enzymes, including catalytic and regulatory subunits of glutamate-cysteine ligase (GCL), glutathione peroxidase (GPX), glutathione reductase (GSR), glutathione S-transferase (GST), as well as reduced glutathione (GSH), and vitamins C and E. Astrocytes also control the supply of energy substrates to the neurons to activate the pentose phosphate glucose utilization pathway that supports glutathione in its reduced state [21]. The NMDAR function is modulated by redox systems through the forming of disulfide bonds in receptor subunits that reduce NMDAR conductivity [22]. Astrocytic abnormalities in schizophrenia include disorders of glutamate reuptake, recycling, and turnover of endogenous NMDAR ligands [23]. The NMDAR hypofunction leads to cortical oxidative stress, GSH deficiency, and decreased activity of the thioredoxin/peroxiredoxin system through transcriptional control of several critical antioxidant genes [24].

Oligodendrocytes, which are necessary for maintaining a high speed of signal transmission through axons and maintaining the metabolism of neurons, are extremely vulnerable to OS's effects. These cells require significant energy costs to maintain and form massive areas of the membrane, stacked in myelin sheets throughout the entire period of postnatal development. Also, myelin itself is a rich source of PUFA. Among all CNS cells, oligodendrocytes have the highest iron content, which is also necessary for the production of myelin [25]. When myelin is damaged, iron is released into the extracellular space and causes the formation of hydroxyl radicals during the transition of Fe^2+^ to Fe^3+^, thereby contributing to the development of OS. This may also lead to the death of microglial cells caused by the absorption of Fe from the intercellular space [26].

Microglia, as the main immunological compartment of the central nervous system performing protective and immunoregulatory functions, is itself a ROS source. They are necessary as the central acting units of extracellular effector and intracellular signaling systems that regulate anti-inflammatory and antioxidant response and the main transcription programs via NF-*κ*B and Nrf2, respectively [27]. However, in schizophrenia, excessive activation of microglia was detected, which leads to an increase in ROS production [28] and the development of neuroinflammation [29].

Different brain regions also differ in their susceptibility to OS. The regions of the brain that are most sensitive to OS are the amygdala, hippocampus, and cerebellar granule cells of the cerebellar cortex. Neurons with different sensitivity to OS can be found in each brain region [30].

The pyramidal neurons of the hippocampus of the adjacent CA1 and CA3 regions are similar in morphology, but not in their sensitivity to OS. A moderate decrease in GSH has been shown to cause a more pronounced OS in the CA1 region [31]. The accumulation of ROS in the CA1 area is accompanied by the significant destruction of neurons, which is observed in the CA3 region to a much lesser extent [32]. When creating an OS model by removing GPX4, it was shown that in the hippocampus, OS causes local neurodegeneration [33].

Neurons in the midbrain, namely the dopaminergic neurons of the pars compacta of substantia nigra (A9) and the adjacent ventral region (A10), also respond differently to OS. Despite the high content of Fe and Cu ions in these regions and the processes of dopamine autooxidation occurring in both parts of the midbrain, A9 neurons are more sensitive to OS effects [6].

Granular neurons in the cerebellum are susceptible to OS. It was shown that the superoxide generated by xanthine oxidase in granular cells induced apoptosis both directly through activation of caspase-3, and indirectly through a violation of the Ca^2+^ balance [34]. In neurons of the cerebral cortex (layers IV-VI), this reaction was almost not observed, so it can be argued that this area is less sensitive to OS [6].

Thus, many signaling and metabolic pathways in brain tissues provoke enhanced ROS formation, which is often exacerbated by the region-specific sensitivity of the brain to OS. In schizophrenia, these features become critical, compounded by the fact that OS plays a significant role in the pathogenesis of the disease, leading to the formation of stable disruptions of the redox balance.

## 3. Altered Redox Balance in Schizophrenia

Now, there is overwhelming evidence of redox imbalance in schizophrenia. This issue is considered thoroughly and in detail in numerous reviews [35–37] and meta-analyses [38–46]. The main markers of redox imbalance in schizophrenia are summarized in Table 1. Regardless of data heterogeneity, the predominance of prooxidant processes and deficiency of the antioxidant system, that is, the state of generalized oxidative stress, are mostly observed in schizophrenia. The main changes in the nonenzymatic antioxidant system consist of decreasing the concentrations of bilirubin, uric acid, ascorbic acid, tocopherol, pyridoxal, folate, and polyunsaturated fatty acids (PUFAs). The data on the reduction of folate and pyridoxal in blood serum of schizophrenia patients were confirmed by meta-analyses [42, 44]. The data on the reduction of PUFAs of the red blood cell membrane in patients treated with antipsychotic medication and antipsychotic-naïve patients were supported by meta-analysis [41] and seem convincing. Besides, the reduction of uric acid in the serum of patients was demonstrated and supported by meta-analysis [40]. Numerous data are indicative of the decreased concentration of reduced glutathione and increased concentration of oxidized glutathione in plasma [47, 48], erythrocytes [49], cerebrospinal fluid [50], and different brain regions [50–52] in first-episode, nonmedicated, medicated, and chronic schizophrenia patients.

The primary markers of disorders in the fermentative antioxidant system in schizophrenia are related to oppositely directed changes in the activity of antioxidant ferments (Table 1). According to the meta-analysis results, the level of activity of erythrocyte superoxide dismutase was reduced in acute relapse of psychosis, drug-naïve first-episode psychosis, stable medicated outpatients, and chronic inpatients [40]. The level of activity of erythrocyte catalase was decreased in the mentioned groups of patients, except for stable medicated outpatients, in whom this level was increased [40]. The level of activity of erythrocyte glutathione peroxidase was reduced in acute relapse of psychosis and chronic schizophrenia patients [40]. However, according to the results of other meta-analyses, the changes in the level of activity appeared to be statistically insignificant [43, 45]. This is explained by small samples of patients, high heterogeneity of groups, and the effect of therapy.

Markers of free radical oxidation products (Table 1) prove the predominance of prooxidant processes in schizophrenia. Numerous data indicate an increase in the concentration of thiobarbituric acid reactive substances (for example, malondialdehyde), lipid peroxides, 4-hydroxynonenal, 3-nitrotyrosine, 8-hydroxy-2-deoxyguanosine, and others. The most reliable proof of predominance of prooxidant processes is the increase of thiobarbituric acid reactive substances in drug-naïve first-episode psychosis, stable medicated outpatients, and chronic inpatients, which is confirmed by the results of meta-analysis [40, 46]. Another remarkable proof of redox imbalance in schizophrenia is the reduction of the total antioxidant status in drug-naïve first-episode psychosis, which is also confirmed by meta-analyses and reviews [35–37, 40, 45].

Only a limited number of studies indicate reductive stress in schizophrenia [53, 54]. Using phosphorus magnetic resonance spectroscopy studies, Kim et al. found a significant decrease in the NAD+/NADH ratio in both first-episode patients and chronic schizophrenia patients [53]. Chouinard et al. replicated this observation and extended it to siblings of schizophrenia patients with first-episode psychosis [54]. A decrease in the NAD+/NADH ratio indicates a shift in redox equilibrium towards a higher recovery potential. The reductive stress condition can lead to a paradoxical increase in mitochondrial ROS production [55]. Thus, both oxidative stress and reductive stress can contribute to oxidative damage in schizophrenia.

## 4. Molecular Mechanisms of Oxidative Stress in the Pathogenesis of Schizophrenia

**Figure 1:**
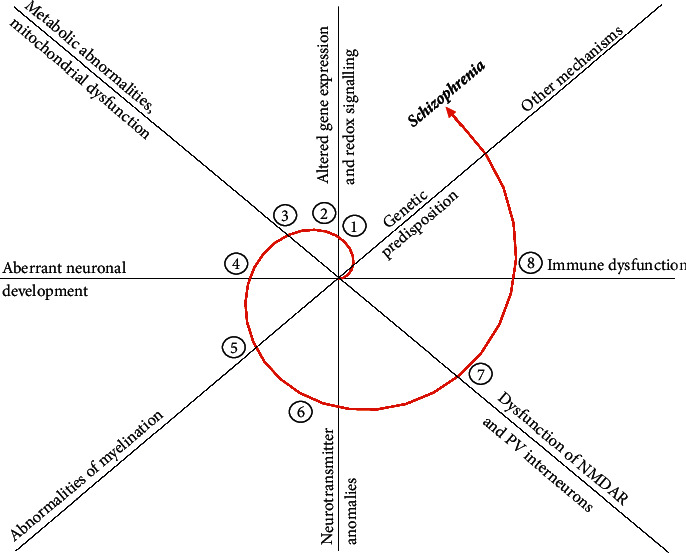
Oxidative stress-related mechanisms of schizophrenia pathogenesis. The involvement of each mechanism increases the likelihood of phenotypic realization and the manifestation of schizophrenia. The red line indicates the probability of developing schizophrenia; various causal mechanisms are plotted along the axes. All of these mechanisms can be involved both together and separately and during different critical periods. Various genetic causes (1) contribute to the increased susceptibility of individuals to oxidative stress. Genetic predisposition due to environmental impact at various critical periods contributes to redox imbalance, which leads to dysregulation of gene expression and redox signaling (2). These changes promote mitochondrial dysfunction and metabolic abnormalities (3). These processes, in turn, contribute to aberrant neuronal development (4) and abnormal myelination (5). These factors promote the neurotransmitter anomalies (6) and dysfunction of parvalbumin-positive interneurons (7). Immune dysfunction (8) also contributes to oxidative imbalance. All these mechanisms ultimately contribute to the manifestation of psychosis and the development of schizophrenia. Abbreviations: PV = parvalbumin; NMDAR = N-methyl-D-aspartate receptor.

The redox imbalance can contribute to the development of schizophrenia at various levels and through various mechanisms. Diverse oxidative stress-associated molecular mechanisms (summarized in Figure 1) involved in the schizophrenia pathogenesis will be discussed in detail below.

### 4.1. Genetic Predisposition to Oxidative Stress in Schizophrenia

Polygenic deterministic predisposition to mental pathology is now proven. Schizophrenia, in particular, falls in the category of multifactorial diseases, whose development is a consequence of intergenic gene-environment impacts and interactions [56, 57]. Regarding schizophrenia, the significance of genetic factors is as high as up to 80%.

According to the literature data, some oxidative stress-related genetic polymorphisms are associated with schizophrenia (reviewed in [58]). First, GSH-related genes, such as glutathione synthesis genes and genes of glutathione-dependent antioxidant ferments, are associated with schizophrenia. In particular, the association with genes of glutamate-cysteine ligase subunits [59–61], being the main rate-limiting enzyme of glutathione synthesis, has been found. At the same time, no association with glutathione synthase genes has been observed in Danish and Swiss populations [61]. Besides, the association of single nucleotide polymorphisms (SNPs) of glutathione S-transferase genes with schizophrenia has been discovered [62–65], whereas no association of SNPs of glutathione peroxidase 1 genes has been revealed [66].

Second, the association of polymorphous variants of genes of antioxidant ferments with schizophrenia has been shown. The association of polymorphisms of manganese superoxide dismutase (Mn-SOD) genes has been demonstrated only for the Turkish sample, with negative reports from Korean, Japanese, and Caucasian samples [58]. In the Russian [67], Polish [68], Japanese [69], and Xhosa [70] populations, the association of Mn-SOD genes with tardive dyskinesia, which is a side effect of antipsychotic medications, has been observed. However, attempts to reproduce these observations in other populations failed [71–75]. No association of polymorphous variants of catalase genes with schizophrenia was found [76–78]. The association with genes of methionine sulfoxide reductase [79, 80], which regulates the activity of the central dopamine degradation ferment—catechol-O-methyl transferase [81], has been observed.

Third, the association of nitric oxide metabolism genes with schizophrenia has been revealed (reviewed in [82]). In particular, the association with genes of nitric oxide synthase 1 and nitric oxide synthase 1 adaptor protein has been shown [82].

Fourth, the association with mitochondrial genes was found [58]. Indeed, the association with the MTND4 gene (ND4 subunit of NADH-ubiquinone reductase) [83] and other genes of mitochondrial DNA was reported [84]. Besides, in nuclear DNA, the association of the DISC1 gene with schizophrenia was discovered [85–87]. This gene mostly expresses in mitochondria [87] and takes part in mitochondrial transport, neuronal axon, and dendrite outgrowth, as well as proliferation, differentiation, and migration of neuronal cells. However, the recent genome-wide association studies (GWASs) with large groups of patients and healthy donors failed to prove the mentioned associations with oxidative stress-related genetic polymorphisms [88–91]. It seems necessary to carry out GWASs with larger samples.

However, the most important result of the GWASs is proof of the polygenic nature of schizophrenia [88–91]. The association with numerous genes, each being a minor contributor to the disease's pathogenesis, has been revealed. According to the tentative estimate, the combination of 6,300 to 10,200 individual SNPs can provoke the development of schizophrenia [90]. Besides, many discovered associations can also be related to other mental diseases [92], which is indicative of some general mechanisms of the development of mental disorders. Also, the results of GWASs suggest that the disturbance of the regulation of gene expression is more significant for the etiopathogenesis of schizophrenia than changes in exonic regions of the genome (and, correspondingly, in protein sequences) because many associations were found beyond protein-coding regions of the genome [89, 90].

### 4.2. Gene Expression Dysregulation, Noncoding RNAs, Environmental Impact, and Oxidative Stress in Schizophrenia

Gene expression dysregulation in different tissues in schizophrenia is confirmed by numerous postmortem studies [93–96]. The transcription studies of the PsychENCODE consortium with the use of RNA-sequencing techniques and state-of-the-art analysis methods are indicative of complex spatiotemporal, sexual, cell-specific alterations in gene expression, splicing, and transcript isoforms levels in the brain of schizophrenia patients [97, 98]. It is noteworthy that the top pathways for diurnal rhythms in prefrontal cortex gene expression that were different in schizophrenia compared to healthy controls are oxidative phosphorylation and mitochondrial dysfunction [99]. The results of proteomic studies also confirm gene expression dysregulation in schizophrenia [100–102]. Prabakaran et al. showed that almost half of the proteins with altered expression were associated with oxidative stress responses and mitochondrial function [103]. Thus, genetic risk factors, along with gene expression dysregulation, form the basis of etiology and pathogenesis of schizophrenia and are associated with redox imbalance.

The available data on gene expression dysregulation in schizophrenia can be explained by dysregulation of transcriptional factors [96], particularly those participating in redox signaling. Indeed, SNPs of PDCD11 gene, coding the NF-*κ*B-binding protein (NFBP) or the protein RRP5 homolog, have shown a statistically significant association with schizophrenia according to the GWAS results [91]. It is known that NFBP specifically binds to p50 and p65 subunits of nuclear factor kappa B (NF-*κ*B) [104]. Besides, the association with TRIM8 (Tripartite Motif Containing 8) gene [91], coding E3 ubiquitin-protein ligase TRIM8, has been shown. E3 ubiquitin-protein ligase is known to potentiate TNF*α*-and IL-1*β*-induced activation of NF-*κ*B [105]. Shotgun proteomic analysis revealed that TRIM3 was upregulated in postmortem dorsolateral prefrontal cortex samples obtained from schizophrenia patients compared to its level in healthy individuals [106]. One of the missense mutations revealed in schizophrenia in TRIM genes is located in the catalytic RING domain. Therefore, this variant may alter ubiquitin ligase activity of this protein [106] and thus disturb NF-*κ*B activation. Accordingly, some GWAS-identified SNPs associated with schizophrenia can affect NF-*κ*B signaling. Taking into account that NF-*κ*B participates in redox signaling, dysregulation of this factor can favor redox imbalance.

Gene expression dysregulation in schizophrenia is also associated with abnormalities in noncoding RNA-mediated regulation [107]. Noncoding RNAs (ncRNAs) are known to take part in redox regulation; moreover, ncRNAs affect ROS generation and ROS affect ncRNA transcription (reviewed in [108]). Among numerous ncRNAs dysregulated in schizophrenia [107], some are related to oxidative stress responses. Indeed, microRNA-30b expression was reduced [109], whereas the expression of microRNA-181a was increased [110] in brain samples of the prefrontal cortex of patients with schizophrenia. One of the target genes of microRNA-30b is CAT (encoding сatalase), and the target gene of microRNA-181a is GPX1 (encoding glutathione peroxidase 1) [108]. In another example, the level of expression of microRNA-146a, which regulates the SOD2 gene (encoding superoxide dismutase 2) expression, was increased in peripheral blood mononuclear cells [111]. Besides, in the systematic review by Smigielski et al., dysregulation of microRNA-34a (mostly upregulation) and microRNA-132 (mixed pattern) was identified in different tissues of patients with schizophrenia [112]. These microRNAs take part in the regulation of nuclear factor erythroid 2-related factor 2 (Nrf2) that regulates the expression of numerous antioxidant proteins [113]. The increase in the level of expression of microRNA favors the silencing of the target gene. In contrast, the reduction of microRNA expression favors the increase of the target gene expression, although some exceptions are possible. Thus, the discovered abnormal level of microRNA expression in schizophrenia can globally affect the redox balance. However, it cannot be excluded that the change in the ncRNA level can also respond to a shift in redox balance [108].

Various environmental insults can contribute to gene expression dysregulation and redox imbalance in schizophrenia. Primarily, inflammation-mediated immune responses are also accompanied by abnormal gene expression, as well as by the oxidative stress. Indeed, animal model studies confirm that lipopolysaccharide- (LPS-) induced maternal immune activation leads to an increase of expression profiles of oxidative stress-related genes and a decrease of expression profiles of critical neurodevelopmental genes in the fetal brain [114]. Other environmental insults associated with oxidative stress and schizophrenia are pre- and postnatal protein malnutrition [115] and hypoxia [116]. Besides, it was shown with the mouse model that prenatal hypovitaminosis D alters the gene expression of several biological pathways, including oxidative phosphorylation and redox balance [117]. Studies in the human population confirm the influence of neonatal vitamin D status on the risk of schizophrenia [118]. Early life adversity, for instance, maternal separation, favors oxidative stress in parvalbumin- (PV-) positive neurons [119] (more on the role of parvalbumin-expressing neurons is discussed further). Prenatal stress also supports oxidative stress and neuronal loss in the rat hippocampus [120]. At the same time, human epidemiological studies confirm that prenatal stress due to grief, famine, and major disasters has effects on vulnerability to schizophrenia [121]. It addition, it was shown that postweaning social isolation disturbs antioxidant defense mechanisms in cortical parvalbumin- (PV-) positive interneurons, supposedly mediated by downregulation of peroxisome proliferator-activated receptor-gamma coactivator 1-alpha (PGC-1*α*), which is a transcriptional coactivator and participates in the regulation of mitochondrial energy metabolism [122]. The oxidative stress after social isolation in rats was caused by increased expression of hypoxia-inducible factor-1*α* (HIF-1*α*) and redox-sensitive transcription factor c-fos. A treatment NOX inhibitor apocynin prevented histopathological and behavioral alterations [123].

Epigenetic mechanisms can serve a connecting link between environmental and genetic factors (reviewed in [112, 124, 125]). The following impairments of epigenetic regulation were found in schizophrenia: aberrant DNA methylation at approximately 100 loci, including genes regulating glutamatergic and GABAergic systems, genes of the stress response, and genes regulating the development of the nervous system, was shown; an alteration in the methylation status of different genes led to a change in symptoms of a disease; increased activity of DNA-methyltransferase 1 in interneurons of the hippocampus and striatum was found; increased levels of methyl group donor–S-adenosylmethionine was revealed in the prefrontal cortex of patients; increased level of homocysteine in blood serum was observed in acute schizophrenia; and histone modifications leading to dysregulation of different genes were found [112].

A considerable part of the epigenetic alterations in schizophrenia can be acquired through numerous environmental factors, and epigenetic changes can affect brain functions throughout the entire life. They can be inherited via epigenetic germline inheritance [91]. Besides, epigenetic modifications may be the molecular basis of the phenotypic heterogeneity of schizophrenia [125].

Thus, genetic susceptibility and gene expression dysregulation caused by abnormal regulation of transcriptional factors, noncoding RNAs, and epigenetic mechanisms favored by environmental insults form the basis of the prooxidant state underlying the pathogenesis of schizophrenia.

### 4.3. Altered Redox Regulation and Redox-Dependent Signaling in Schizophrenia

Maintenance of redox balance is essential for the cell and the entire organism. The redox regulation is controlled by different mechanisms [113] and is closely related to redox signaling [126]. Accurate redox regulation is necessary for cellular signaling because reactive oxygen and nitrogen species (ROS and RNS) participate in numerous signaling pathways [127, 128]. ROS or RNS in physiological concentrations take part in cell-signaling mechanisms, but in high levels, they favor oxidative stress [127, 128]. In the broad sense of the term, redox signaling is signaling processes accompanied by electron transfer reactions in which ROS and RNS or reductive equivalents are involved [129].

Oxidation and reduction of cysteine residues in transducing signal proteins are assumed to be the fundamental mechanisms by which ROS and RNS integrate into cellular signal transduction pathways [126]. Many signal events are accompanied by the generation of ROS serving as secondary messengers, thus leading to oxidative modifications of cysteine [127, 128]. Oxidative posttranslational modifications of cysteine residues in proteins under the effect of ROS include oxidation of cysteine thiols to sulfenic acid (SOH), sulfinic acid (SO_2_H), and irreversible sulfonic acid (SO_3_H) [130]. RNS interacts with cysteine thiols with the formation of reversible *S*-nitrosothiol [130]. The following reaction consists of intramolecular disulfide bond formation or conjugation with GSH (S-glutathionylation) [130]. S-glutathionylation can occur chemically or fermentatively via glutathione S-transferase (GST), peroxiredoxins, and occasionally glutaredoxins [130]. Reversing the oxidized cysteine residues in the sulfenic acid state occurs through the thioredoxin or GSH-dependent pathway [131]. Reversing the sulfinic acid state requires sulfiredoxin [131]. The reductive cellular environment (GSH) or catalysis by glutaredoxins remove protein-bound GSH and restore the protein cysteine [130]. Thus, glutathione is an essential molecule in signal transduction regulation. Oxidative stress and GSH deficiency, which are observed in schizophrenia [132, 133], can break the oxidation and reduction cycles of the cysteine residues, thereby disrupting redox signaling.

Many protein tyrosine phosphatases (PTPs) participating in signal cascades are direct targets of ROS and RNS [131]. Generation of ROS in response to, for example, receptor activation leads to inactivation of PTPs, thus leading to an increase of phosphorylation of numerous kinase targets, which is a necessary event for downstream signaling [131]. ROS inactivate not only PTPs but also dual-specificity phosphatases (for example, PTEN (phosphatase and tensin homolog deleted on chromosome 10)), low-molecular-weight PTPs, and cell cycle phosphatase [134]. Besides, ROS activate indirectly mitogen-activated protein kinases (MAPK), in particular, apoptosis signal-regulated kinase 1 (ASK1), through cysteine oxidation of thioredoxin, which directly inhibits its kinase activity [127]. The prooxidant state in schizophrenia can favor abnormal signaling.

Redox imbalance can also influence transcriptional factors. There are multiple ROS sensors and pathways involved in the redox-gene transcription regulation, in particular, Nrf2 (nuclear factor erythroid 2 related factor 2), NF-*κ*B (nuclear factor kappa B), FoxO (forkhead box class O), AP-1 (activator protein 1), CREB (cAMP response element-binding protein), HSF1 (heat shock factor 1), TP53 (tumor protein p53), HIF-1 (hypoxia-inducible factor 1-alpha), SP1 (specificity protein 1), and other proteins [113].

The Nrf2-Keap1 (Kelch-like ECH-associated protein 1) pathway is one of the primary regulators of responses to oxidative stress [113]. Under normal conditions, Nrf2 is inactivated by Keap1-mediated ubiquitination and subsequent proteasomal degradation. Sulfhydryl groups of Keap1 act as ROS sensors, and their oxidation in the presence of ROS leads to nuclear translocation of Nrf2 and increased expression of antioxidant genes [113]. Transcription factor Nrf2 has shown the ability, both in *in vitro* and in *in vivo* experiments, to activate a series of vitagenes including (Hsp) Hsp32, Hsp70, and thioredoxin, conferring protection against oxidative stress, and contributing to establish a cytoprotective state in inflammation and neurodegenerative disorders [135, 136]. In the normal state, these pathways are considered as hormetic mechanisms of adaptive cellular stress response [137, 138]. In schizophrenia patients under conditions of systemic oxidative stress, the decreased Nrf2 expression in peripheral blood lymphocytes was discovered [139]. Besides, expressions of Nrf2 and Keap1 proteins in the parietal cortex from brain samples of schizophrenia patients were lower than those of healthy individuals [140].

NF-*κ*B takes part not only in the regulation of genes of immune response, development, proliferation, and apoptosis but also in redox regulation [141]. ROS promote dissociation of inhibitory proteins and activation of NF-*κ*B [113]. The increases in mRNA levels for NF-*κ*B family members, NF-*κ*B activation receptors, kinases, and inhibitor protein (I*κ*B*α*) were found in the prefrontal cortex of schizophrenia patients [142].

The forkhead box class O (FoxO) family of transcription factors are critical regulators of the expression of genes involved in cellular oxidative stress response, ROS detoxification, DNA repair, energy homeostasis, and glucose metabolism (reviewed in [143]). FoxO transcription factors regulate numerous genes coding for intra- and extracellular antioxidant proteins such as Mn-SOD and Cu,Zn-SOD, peroxiredoxin-3 and peroxiredoxin-5, mitochondrial thioredoxin (Trx2) and mitochondrial thioredoxin reductase, glutathione peroxidase 1, and selenoprotein P [143]. Several protein kinases such as protein kinase B (Akt), extracellular signal-regulated kinase (ERK), p38 mitogen-activated protein kinases (p38MAPK), and c-Jun N-terminal kinase (JNK) phosphorylate FoxO transcription factors in response to elevated levels of ROS and upon exposure of cells to stressful stimuli [143]. Moreover, the effects are different depending on protein kinases; for instance, phosphorylation by Akt usually inactivates FoxO1a, FoxO3a, FoxO4, and FoxO6 proteins, whereas JNK activates FoxO4 and inactivates FoxO3a [143]. It was shown in the mouse model that behavioral stress can activate FoxO3a in the cerebral cortex through inactivation of Akt and is accompanied by activation of glycogen synthase kinase-3*β* (GSK3*β*) [144]. It was shown that the expression and activity of AKT1 were reduced in lymphocytes and in the frontal cortex and hippocampus of individuals with schizophrenia [145]. In another study, the reduction of expression of Akt and other Akt-mTOR signaling pathway proteins in the dorsolateral prefrontal cortex in schizophrenia was demonstrated [146]. The expression of JNK1 and JNK2 was decreased in the anterior cingulate cortex of schizophrenia patients, while the expression of ERK1/2 or p38 was unchanged [147]. Besides, different antipsychotics and psychoactive substances affect dopamine-associated behaviors through modulation of the Akt/GSK3*β* signaling pathway [148]. It is also known that clozapine can regulate the activity of the Akt/FoxO3a signaling pathway via phosphorylation of Akt and FoxO3a [149]. Thus, alteration of the activity of protein kinases, in particular, as a result of antipsychotic treatment, can modulate FoxO activities. This opens new ways of using inhibitors of protein kinases in the modulation of redox-regulated transcription factors.

Another mechanism of regulation of FoxO activities consists of acetylation and ubiquitination of lysine residues. Histone acetyltransferases and deacetylases catalyze reversible lysine acetylation. The ubiquitination of FoxOs proceeds by ubiquitin-protein ligases and promotes proteasomal degradation of FoxOs. Oxidative stress caused by increased intracellular levels of ROS and RNS, particularly H_2_O_2_, has been identified as a critical mediator of the state of acetylation and ubiquitination of FoxOs [143]. Indeed, exogenous H_2_O_2_ induces the formation of heterodimers between coactivators (p300 and CBP (CREB-binding protein) acetyltransferases) and FoxO4 through intermolecular disulfide bridges between redox-sensitive cysteine residues and stimulates acetylation of FoxO4 [150]. Brunet et al. have shown that NAD-dependent deacetylase sirtuin-1 (SIRT1) is a crucial regulator of the activity of FoxOs [151]. The activity of SIRT1 depends on the redox state of a cell, is regulated by the NAD+/NADH ratio, and is activated at a restriction of reduction equivalents [143]. SIRT1 is associated with the depression behavior in the mouse model [152] and with depressive symptoms in schizophrenia patients [153]. It is noticeable that there exist medicines increasing SIRT1 activity, for example, resveratrol [152] or salvianolic acid B [154], which can promote correction of redox imbalance through an increase in activity of Nrf2 [154] and, likely, FoxO, as well as through a decrease in the activity of NF-*κ*B. However, an increase in the activity of SIRT1 in the nucleus accumbens may favor anxiety- and depression-like behaviors [152].

The transcriptional coactivator PGC-1*α* (peroxisome proliferator-activated receptor-gamma coactivator 1*α*) is an upstream regulator of energy metabolism and mitochondrial biogenesis. PGC-1*α* has been shown to regulate FoxO transcription factor activity in various cells [143]. GWASs have identified that PGC-1*α* is one of the candidate genes for schizophrenia [155]. PGC-1*α* knockout mice presented some characteristic features of schizophrenia [155]. Besides, PGC-1*α* gene deletion delayed maturation of PV interneurons, including their perineuronal nets [155]. As stated above, postweaning social isolation leads to the downregulation of PGC-1*α* in mice [122]. PGC-1*α*-dependent transcripts in postmortem cortical tissue from schizophrenia patients were reduced and accompanied by a decrease in expression of Nrf1 as well as PV [156]. The data presented relate redox imbalance and altered redox regulation to the pathology of PV interneurons [156] (more on PV neuron pathology will be discussed below).

Thus, there are some data on the altered redox regulation and signaling in schizophrenia, which is related to prooxidant processes, glutathione deficiency, and impaired expression of transcriptional factors and multiple ROS sensors.

### 4.4. Effect of Oxidative Stress on Neuronal Development

The dysontogenetic hypothesis of schizophrenia was first formulated more than three decades ago [157, 158]. Today, it is proved by a wide range of studies whose results are indicative of neuroanatomical and cytoarchitecture brain disorders, such as disorders in the structure of synapsis and mediator systems, decrease of oligodendrocytes, and reduction of myelination. Many factors affecting the pre- and postnatal periods of development are considered as causes for these disorders. The prenatal causes include in utero exposure to viral and bacterial infections during pregnancy, maternal chronic diseases, severe nutritional disturbances of a mother during pregnancy, obstetric complications, and influence of alcohol, narcotic substances, and pharmaceutical products on the fetus. The main postnatal factors are social deprivation and psychogenic stress at an early age [159, 160].

Several studies are confirming the relation of schizophrenia to such viral diseases as influenza, herpes (Herpes Simplex Virus Type 2 (HSV-2)), and rubella [161]. The response of the maternal organism to an infectious disease leads to the activation of cytokines, which, in turn, gives rise to the risk of psychotic disorder. Buka et al. have found that the increased level of TNF*α* in mothers with infectious disease in the third trimester of pregnancy leads to an eightfold increase in the risk of psychotic disorder in the child as an adult [162]. Maternal TNF*α* penetrates through the placental barrier into fetal CNS [163] and leads to ROS generation through activation of NADPH oxidase [164]. The studies in animals show that cytokines penetrating through the fetal blood-brain barrier (BBB) significantly affect the survival and differentiation of neurons [165]. The paper by Simões et al. reports a significant increase in IL-1*β*, IL-6, and TNF*αβ* levels in the offspring brain in response to maternal cytokines [166]. The inflammatory response leads to an increase in ROS generation by endothelial cells, and, consequently, to disruption of BBB permeability [167]. In addition to the disruption of BBB and placental barrier permeability, cytokines in the brain regulate the expression of major histocompatibility complex I (MHC I), coordinating synaptic pruning [168, 169]. Postmortem examination of the brain in schizophrenia patients shows that synaptic proteins interact with the complement system and other immunological pathways, causing changes in glial structures and synapse elimination [170].

Activation of cytokines in the maternal organism not only can be caused by the effect of an infectious agent but also can be induced by the action of ethanol on the fetus. The studies of González-Quintela et al. have demonstrated that the expression of IL-1*α*, IL-6, and TNF*α* can increase after single-dose administration of 60 g ethanol [171]. It has also been shown that chronic alcohol consumption increases the transcription of TNF*α*, IL-1*β*, and toll-like receptor 4 (TLR4) in the brain cortex and hypothalamus [172]. In the experiment with rats, at the intraperitoneal injection of ethanol in the concentration of 4 g/kg, a considerable increase of IL-6 expression in the hippocampus, paraventricular nucleus of the hypothalamus, and tonsil was observed [173]. Ethanol significantly affects neural membranes and synaptic contacts between developing neurons. In a fetus of an alcoholic mother at 9–12 weeks of pregnancy, the slower development of synapses, the shorter length of the postsynaptic density, and the smaller perimeter and area of the presynaptic terminal were observed. Ethanol-induced oxidative modification of proteins and lipids of cell membranes can be one of the mechanisms of the toxic effect of ethanol at neurogenesis [174].

At the chronic effect of ethanol, the amount of ROS and NO in the brain increases through induction of NADPH oxidase and NO synthase under the exposure to glial cytochrome P450-2E1 (CYP2E1) [175, 176]. In the study with mouse models, it was shown that an ethanol-induced disturbance of the redox homeostasis leads to activation of microglia by the M1 phenotype [177, 178]. Activation by the M1 phenotype leads to the secretion of proinflammatory cytokines and chemokines and ROS production.

The increased synthesis of proinflammatory cytokines is observed in obese pregnant women. It was revealed that the body mass index correlates directly with the concentration of proinflammatory cytokines in the mother and activation of proinflammatory pathways in the placenta [179]. In animal models, it was shown that obesity during pregnancy is related to systemic and placental inflammation, oxidative stress, and antioxidant deficit in the bodies of the mother and the fetus [180]. Edlow et al. have demonstrated that fetal expression of apolipoprotein D (APOD) gene was nine times higher in the case of an obese mother [181]. The increased APOD expression, in turn, was found in schizophrenia patients [182].

In addition to obesity, gestational diabetes and pregestational diabetes are possible factors of increased ROS generation [183, 184]. Under conditions of excess glucose in the mother's blood, the increased production of the superoxide anion radical (O_2_^-•^) is observed [185]. Consequently, the disturbance of glucose exchange in the prenatal period leads to changes in the structure and functions of the plasmatic membrane of neurons and the receptor apparatus of neurotransmitters, thus increasing the risk of schizophrenia at adult age. The decreased placental levels of arachidonic acid (AA) and docosahexaenoic acid (DHA) were found in mothers and children with gestational diabetes [186]. These factors have a maximally unfavorable effect on the development of the child's brain: polyunsaturated fatty acids take part in synaptogenesis and synthesis of neuromodulators and prevent the synthesis of signal molecules associated with Alzheimer's disease and schizophrenia [187]. In postmortem studies, it was shown that schizophrenia patients had decreased AA and DHA levels in the frontal cortex [188]. The reduced level of polyunsaturated fatty acids is also assigned to the disruption of synaptic transmission of dopamine and GABA, which plays an essential role in the pathogenesis of schizophrenia [189, 190].

Recent studies demonstrate the relationship between the psychosocial stress in the postnatal period and the oxidative stress [191, 192]. It was assumed that the disturbance of the regulation of the hypothalamus-pituitary-adrenal axis could mediate the relationship between childhood trauma and psychosis. Colaianna et al. demonstrate that psychosocial stress leads to the increased ROS generation by NADPH oxidase 2 (NOX2) in the hypothalamus [193]. The oxidative stress in the hypothalamus disturbs the functions of the hypothalamus-pituitary-adrenal axis, contributing to psychosis development [194, 195]. Disorder in the development of the nervous system assumes that the prolonged exposure to stressors can cause the increased release of glucocorticoids, which stimulate the dopaminergic activity, thus increasing the risk of psychosis [196]. As is known, the increased dopamine synthesis is related to symptom severity at the prodromal stage. Besides, dopamine hyperactivation appears in schizophrenia patients in the acute stage and after psychological stress [196]. Glucocorticoid neurotoxicity favors the loss in hippocampus volume, which is observed in schizophrenia patients even at early stages [197].

Many investigators indicate that disorders of the prenatal brain development are related to the activation of oxidative stress. The activation of maternal immune reactions leads to the increased synthesis of cytokines and ROS, thus mediating inflammatory responses in the fetal brain. In addition to inflammatory responses, ROS affects the cell membranes of neurons, leading to the disturbance of the neurotransmission of dopamine, glutamate, and GABA, which are the main neuromediators involved in the pathogenesis of schizophrenia. Thus, maternal infectious diseases, obesity, diabetes, alcohol ingestion, and other factors lead to the development of oxidative stress in the fetal brain, which can be a predictor of schizophrenia development at adult age, in which the process of synaptic pruning in adolescence may be the starting point.

### 4.5. Metabolic Abnormalities and Mitochondrial Dysfunction

Mitochondria are not only the leading energy supplier of the cell but are also actively involved in other critical physiological processes, including redox signaling, calcium homeostasis, cellular differentiation, and apoptotic cell death. The role of mitochondria is especially crucial for the development and functioning of the nervous system. Mitochondria are involved in the regulation of neuronal differentiation, neuroplasticity, axogenesis, dendritogenesis, and the release of neurotransmitters by generating adenosine triphosphate (ATP) and regulating subcellular calcium concentration and redox homeostasis [198]. Mitochondrial dysfunction and decreased ATP production lead to the disruption of the transmembrane gradient and intracellular calcium buffering as well as enhancing ROS production [199].

Ample evidence has been accumulated, indicating a multifaceted mitochondrial dysfunction in schizophrenia (reviewed in [198, 200, 201]). Primarily, morphological and functional abnormalities of mitochondria in schizophrenia were detected. An electron microscopic study of the postmortem brain tissues of patients with schizophrenia showed a significant decrease in the number and density of mitochondria of oligodendroglial cells in the prefrontal cortex and caudate nucleus [202]. Besides, the functional activities of complexes IV and I+III of the mitochondrial electron transport chain were reduced in some areas of postmortem cortex tissues in patients with schizophrenia [203]. It is known that antipsychotic treatment reduces the activity of complex I in mitochondria [199]. In vivo imaging studies also confirm the functional changes in mitochondria in schizophrenia. The decreased metabolism in the frontal lobe, which was manifested by a decrease in creatine kinase, intracellular pH, and the concentration of macroergic compounds, was found in patients with schizophrenia [204]. Besides, increased ATP levels in white matter and decreased ATP levels in gray matter were found in the frontotemporal-striatal region in first-episode schizophrenia patients [205]. Metabolomics studies also confirmed metabolic abnormalities in schizophrenia [206].

These changes may be associated with impaired gene expression. Indeed, the decreased expression of numerous mitochondria-related genes (encoding, for example, NADH-ubiquinone oxidoreductase core subunits V1, V2, and S1 and cytochrome c oxidase) was observed in postmortem brain tissues of patients with schizophrenia in smaller-scale studies [201]. As stated above, transcriptomic and proteomic studies in large samples also confirmed the impaired expression of mitochondrial genes in brain tissues [98, 103, 207]. Remarkably, altered transcripts evaluated in parvalbumin- (PV-) containing interneurons were enriched for pathways involved in mitochondrial function [207]. A meta-analysis of microarray studies assessing gene coexpression network modules in the prefrontal cortex of schizophrenia patients showed that oxidative phosphorylation, myelination, and immune function are some of the most downregulated enriched modules [208]. A recent meta-analysis considering the gene expression, transcript isoform expression, local splicing, and coexpression network modules identified cell-specific dysregulation, including protein-coding, noncoding, splicing, and isoform-level changes in brain samples from individuals with schizophrenia, autism spectrum disorder, and bipolar disorder [98]. Among the confirmed differentially expressed genes, transcripts, and splicing isoforms in patients with schizophrenia, mitochondrial genes were also found [98]. The downregulation of coexpressed gene modules associated with mitochondria in a larger sample of schizophrenia patients was also shown [209].

Evidence also includes the genetic association of mitochondrial genes with schizophrenia. Among the 350 genes within 108 schizophrenia loci identified by GWAS [91], 22 are related to mitochondrial function [201]. For instance, the association of schizophrenia with the USMG5 gene encoding a small subunit of the mitochondrial ATP synthase (complex V), as well as with the SFXN2 gene encoding sideroflexin-2, which is involved in iron metabolism in mitochondria, has been shown [91].

Various animal models have shown that mitochondrial dysfunction leads to neurobehavioral abnormalities (reviewed in [201]). Ablation of the transcriptional coactivator PGC-1*α* reduced the expression of the Ca-binding protein parvalbumin (PV) in the GABAergic interneurons and also disrupted evoked synaptic responses in mice [210]. As stated above, PGC-1*α* is the crucial regulator of mitochondrial biogenesis, including through the regulation of the activity of redox regulating transcription factors FoxOs. Thus, the impaired activity of PGC-1*α* is associated with altered redox regulation and mitochondrial dysfunction, which leads to the pathology of PV interneurons. Besides, the ablation of the cox10 gene in PV interneurons of mice resulted in progressive loss of cytochrome oxidase, which is a terminal enzyme of the electron transfer chain, and was accompanied by an excitation/inhibition imbalance, as well as behavioral alterations similar to schizophrenia [211]. Knockdown of Disrupted-In-Schizophrenia-1 (DISC1) or expression of a dominant-negative C-terminal truncated DISC1 led to a decrease in glucose transporter 4, oxidative phosphorylation, and glycolysis as well as diminished lactate production in mouse astrocytes [212]. These changes were accompanied by altered affective behavior and impaired spatial memory, while lactate treatment rescued these anomalies [212]. Hemizygous deletions of 22q11DS genes were accompanied by haploinsufficiency of mitochondrial large ribosomal subunit protein 40 and led to dysregulation of short-term potentiation via impaired calcium homeostasis in mitochondria [213]. The knockdown of Txnrd2, a 22q11 gene essential for ROS detoxification in brain mitochondria, led to the disruption of the axon and dendrite growth and violated mitochondrial and synaptic integrity in projection neurons; however, antioxidant treatment eliminated these alterations [214]. Therefore, Txnrd2-mediated oxidative stress led to cortical underconnectivity impairment and cognitive deficits [214]. Furthermore, the transfer of isolated mitochondria in induced pluripotent stem cells leads to increased mitochondrial function and improved differentiation into glutamatergic neurons [215]. Injection of isolated mitochondria into the cerebral cortex of adolescent rats in the maternal immune activation model prevents mitochondrial deficiency and behavioral abnormalities at adulthood [215]. These observations link mitochondrial dysfunction, immune disturbances, and impaired neuronal development.

Thus, mitochondrial dysfunction is accompanied by oxidative stress, functional anomalies in neuronal cells, and behavioral alterations and is a prerequisite for the development of schizophrenia.

### 4.6. Oxidative Stress and Abnormal Myelination

Myelination of the human brain proceeds actively in the postnatal period, but the myelin content in the brain peaks at middle age, which coincides in time with the massive remodeling of synaptic contacts in the cerebral cortex [216]. The excess prenatal formation of axons and considerable reduction of axon amount in the postnatal period [217] are compensated by the vast increase of subcortical myelination of residual axons, which are practically absent at birth but grow to up to 25% of the adult human brain volume [218]. These processes reflect the brain formation with gain in experience and optimization of information processes through myelination [219].

Myelin is produced by mature oligodendrocytes (OLs) formed from oligodendrocyte precursor cells (OPCs) in the infantile and adult brains. Neuronal activity can instruct OPCs to divide and mature and stimulate myelin sheath production by OLs [220], leading to increased myelination and improved behavioral performance [221]. Cortical-subcortical white matter (WM) pathways achieve maturation peaks at the age of 23 to 39. This data indicate that WM maturation in frontal regions continues at the time most characteristic of schizophrenia manifestation [222].

Several pathways regulate OL differentiation and myelination in the CNS [223]. The phosphatidylinositol-3-phosphate kinase (PI3K)/Akt/mTOR pathway controls the initiation of myelination in the CNS through mTORC1 signaling [224]. In conjunction, glycogen synthase kinase 3*β* (GSK3*β*) signaling regulates OL differentiation [225]. Moreover, extracellular signal-regulated kinases-1 and -2 (ERK1/2), the downstream mediators of the mitogen-activated protein kinase (MAPK) pathway, regulate myelin growth and maintain the integrity of myelinated axons [226]. These MEK/ERK1/2-MAPK-mediated functions are mostly independent of mTORC1 [227].

Oligodendrocytes can be damaged at the stage of their differentiation from precursors and at the stage of a mature cell. For example, 15-20% of changes in the redox balance in the OPCs, which are extremely sensitive to oxidative stress, may already affect signaling pathways [228]. Factors that cause high vulnerability to oxidative stress include exceptionally high amounts of ROS (six times higher) in OPCs and OLs, three times lower glutathione concentration, and 20-fold higher free-iron levels as compared with astrocytes [229]. These processes promote hypomyelination in the prefrontal cortex and hippocampus.

The decreased volume of cortical grey matter at schizophrenia cannot be fully explained by the loss of synaptic contacts or changes in the microcirculatory bloodstream. Uranova et al. have performed the electron-microscope morphometric study of myelinated fibers in the prefrontal cortex, caudatum, and hippocampus of schizophrenia patients [230]. Local destruction of myelin sheaths and atrophy of axons have been revealed in all the examined brain structures. Besides, the decreased density of WM oligodendrocytes in the frontal cortex was also shown [231]. It is known that uncompensated loss of myelin at schizophrenia forms immediately after the first episode and increases in the course of disease [232, 233]. The OPC dysfunction coincides with the relatively late start of myelination of the prefrontal cortex, causing hypomyelination and distorting connectivity in this part of the brain. It is also assumed that the disturbance of OPCs can affect hypomyelination in the hippocampus; this is confirmed by the results obtained with Gclm-KO mice [234] and the results of postmortem studies of patients with schizophrenia [235, 236].

Unlike astrocytes, which have powerful antioxidant defense mechanisms [237], oligodendrocytes are sensitive to hypoxia and oxidative stress, especially during the terminal phase of differentiation and formation of myelin sheaths. During CNS maturation, the resistance of OLs to oxidative damage increases due to the elevated glutathione levels [238], the transition from the oxidative to the glycolytic mechanism [239], the rise of the longevity of myelin proteins [240], and metabolic support of axons by myelin sheathes [241].

In the context of glutathione deficiency, an increase in the ROS level resulting from a high metabolic rate in the mitochondria of OLs leads to hyperstimulation of the AMP-activated protein kinase (AMPK), which activates the tuberous sclerosis 1/2 complex. This complex prevents the activation of the rapamycin (mTOR)-P70S6K pathway, which leads to OPC proliferation arrest, apoptosis, and hypomyelination [242].

The death of mature oligodendrocytes also promotes neuroinflammation and oxidative stress stimulation. As mentioned above, OLs are rich in iron ions. Iron released by oligodendrocytes is accumulated in macrophages and microglia, which can release iron into the intercellular space, disturbing the integrity of axons [243]. These processes can also stimulate neuronal cell death through ferroptosis [244]. Thus, the wavelike release of iron stimulates the processes of neurodegeneration together with inflammation [245].

Thus, abnormal myelination and dysfunction of the white and gray matter of the brain have been identified in schizophrenia. The molecular mechanisms underlying these changes remain unclear, but undoubtedly oxidative stress also contributes to these processes.

### 4.7. Immune Dysfunction and Oxidative Stress

A growing body of evidence suggests that an “immunooxidative” pathway, including oxidative stress, mitochondrial dysfunction, neuroinflammation, and cell-mediated immune response may contribute to disruptions in brain activity in schizophrenia [53]. Inflammation and increased oxidative stress could constitute a common pathway between early genetic and environmental factors (such as prenatal infections, obstetric complications, hypoxia, or stress during pregnancy) and psychosis [246, 247]. In the immune system, ROS and H_2_O_2_ are not the only products of peripheral and tissue macrophages. Still, they have a physiological role in signaling cascades that controls activation, migration, and differentiation of immune cells [248].

#### 4.7.1. Microglia and Oxidative Stress

An enormous array of sensitive receptors in microglia control the activation status of these immunological cells [249]. Signaling pathways of these receptors are associated with NADPH oxidase (NOX) expression and ROS generation. Thus, activated microglia and astrocytes can become sources of oxidants by activating NOX enzyme cascades; producing interleukins; and releasing glutamate, quinolinic, and arachidonic acid, which may all contribute to neuron damage [131]. Excessive activation of microglia can lead to the neuroinflammation that accompanies many forms of acute or chronic neuropathology, including schizophrenia.

Microglia, as the resident immune macrophages of the CNS, express high levels of superoxide-producing NADPH oxidases (NOX). The primary function of the members of the NOX family is to generate reactive oxygen species (ROS) or H_2_O_2_ that are important in maintaining cell homeostasis through the regulation of crucial redox-dependent pathways [131]. Overproduction of oxidants leads to an excess signal in strength or in time, both of which have pathological effects and cause oxidative stress. In pathological conditions, ROS production by microglia is considered a major cause of neuronal dysfunction [250] through direct oxidative damage to neuronal macromolecules [251] or derangement of neuronal redox signaling circuits.

Initial molecular signaling pathways underlying microglial activation includes activation of toll-like receptors (TLRs), cytokine receptors, complement receptor 3, CD36 receptors, ionotropic and metabotropic purinergic receptors, and neurotransmitter receptors [248]. Microglia express most TLRs, and their expression levels are altered by microglial activation [252]. Signaling through TLRs is essential for priming (hyperresponsiveness) of NOX2 in several ways.

TLR signaling activates IL-1 receptor-associated kinase 4 (IRAK4) in the MyD88-dependent TLR signaling axis. IRAK4 primes NOX and phosphorylates p47phox on several residues to activate the oxidase directly [251]. Microglia cells can have two phenotypes: the proinflammatory classic M1 phenotype, which is associated with anti-inflammatory cytokine production, antigen-presenting properties, and ROS production, and the M2 anti-inflammatory alternative phenotype possessing anti-inflammatory and immunoregulatory activity [253]. Treatment with LPS combined with the proinflammatory cytokine interferon-*γ* (IFN-*γ*) is frequently used to induce and study the M1 type of microglial activation in vitro. These factors activate toll-like receptor 4 (TLR4, also called CD14) and the IFN-*γ* receptor and induce the recruitment of cytosolic phox proteins to the membrane by the phosphorylation of p47phox (neutrophil cytosolic factor 1, NCF1) [254].

TLR engagement also leads to the activation of the GTP/GDP exchange factor VAV, which mediates nucleotide exchange on Rac1, a catalytic subunit of the NOX complex. In addition, Rac1 activated downstream of TLRs also activates p38MAPK (p38 mitogen-activated protein kinases), which may participate in the mobilization of p47phox [255].

Also, it has been found that TLRs depend on NOX activity. NOX-derived ROS directly regulate the partitioning of TLRs to lipid rafts in the membrane [256] or promote the assembly of signaling complexes, which are required for efficient signaling [257].

LPS stimulation of NOX2 activity in microglia may occur through binding with complement receptor 3 (CR3, MAC1, also called CD11b/CD18) [258]. CR3 acts as a phagocytic receptor for C3b/iC3b-opsonized targets including endogenous targets such as synapses [259] and neurites [260]. CR3 ligation leads to direct NOX2 activation as well as through tyrosine-based activation of receptors DAP12 (TYRO protein tyrosine kinase-binding protein) or Fc*γ*Rs (Fc-gamma receptors) [261].

Ionotropic P2X and metabotropic P2Y purinergic receptors are important for the regulation of the microglial actin cytoskeleton [262]. Stimulation of the ionotropic P2X7 receptor by ATP, together with an increase in intracellular calcium, induces ROS production and release in microglia. Increased NOX activity is implemented through ERK1/2-dependent (mitogen-activated protein kinase 3) [263], p38MAPK-dependent, and PI3K-dependent (phosphoinositide 3-kinase) pathways [264].

Microglia express a large number of neurotransmitter receptors. In turn, disruptions of the neurotransmitter environment significantly affect the activation state of microglia and neuronal integrity. NOX activation could be induced by agonists of glutamate metabotropic (mGlu3 and group III), GABAA, and purinergic P2X7 or mGlu5 receptors on the rodent BV2 microglial cell line [263]. A complementary study revealed that activation of microglia in vitro, following NMDA receptor stimulation, was accompanied by secretion of ROS that was toxic to neurons [265].

Furthermore, as in the case of TLR signaling or CR3 ligation in macrophages, NOX2-derived oxidants are implicated in the redox regulation of signaling pathways, in which ROS and H_2_O_2_ act as second messengers in cytokine responses [266]. H_2_O_2_ activates mitogen-activated protein kinase (MAPK) cascades, partly through oxidation of catalytic cysteines on MAPK-inactivating phosphatases [267], and induces nuclear factor kappa B (NF-*κ*B) translocation from the cytosol to the nucleus [268]. The initiation of NF-*κ*B-dependent gene transcription promotes the production of proinflammatory mediators. These include cell adhesion molecules ICAM and VCAM, ROS-producing enzymes like iNOS and NOX2, and cytokines IL-6, IL-8, and, importantly, TNF [269, 270]. Taking into account that TNF and ROS may induce NF-*κ*B-dependent gene transcription, this potentially results in an amplification loop of TNF and ROS signaling [269].

Additionally, the release of cytokines or gliotransmitters by activated astroglia and microglia controls BBB permeability in brain pathologies associated with excessive angiogenesis, cerebrovascular remodeling, and blood-brain barrier-mediated neuroinflammation also observed in schizophrenia [253].

#### 4.7.2. Cytokines and Oxidative Stress

The role of cytokines in the pathogenesis of schizophrenia has been demonstrated in many works [249, 271]. The study of the relationship between the level of cytokines and oxidative markers in schizophrenia using the Bradford Hill criteria to establish a causal relationship confirmed their pathogenic role [272]. A high level of proinflammatory cytokines is associated with a high probability of developing schizophrenia among people at risk compared with people at risk but with a low content of proinflammatory cytokines. According to many studies, the severity of clinical symptoms correlates with the level of cytokines [43].

The study of proinflammatory and oxidative markers in patients with schizophrenia revealed increased levels of proinflammatory cytokines (i.e., IL-1-*β*, IL-6, IL-12, IFN-*γ*, TNF-alpha, tumor growth factor-*β*) and reduced levels of antioxidants (total antioxidant status, catalase, plasma nitrites, and superoxide dismutase) in patients with the first episode of schizophrenia [273].

There are several suggested ways by which proinflammatory cytokines can contribute to schizophrenia development [272]:

1. Inflammation-associated activation of IDO (indolamine 2,3-dioxygenase) enhancing the production of kynurenine metabolites 3-hydroxykynurenine and 3-hydroxyanthranilic acid, both of which are potent generators of radical oxygen species [274]. Thus, it can be proposed that inflammation contributes to neurodegeneration by converting oxidative stress to excitotoxic stress in the context of IDO activation [275]. Kynurenic acid also contributes to the hyperactivation of glutamatergic neurotransmission, which is considered one of the mechanisms for the development of positive symptoms in schizophrenia by itself [273]
2. Proinflammatory cytokines activate microglia, promoting the development of neuroinflammation and oxidative stress [276]. There was a significant increase in the levels of TNF*α*, IL-1*β*, and IL-6 and a decrease in the levels of IFN-*γ* in patients with schizophrenia [249]. Lipid peroxides were elevated in serum, while total sulfhydryl levels were decreased. Superoxide dismutase and glutathione peroxidase were reduced, while the activities of catalase, glutathione reductase, and myeloperoxidase were found to be elevated [275]. Activated microglia are a source of oxidants, which lead to the development of oxidative stress and damage to the membranes of neurons, as described above
3. Proinflammatory cytokines may disturb neurodevelopment (particularly when there is a prenatal inflammation), increasing the risk of psychosis [277]. Activation of the IL-6/Nox2 pathway in schizophrenia leads to the loss of the GABAergic phenotype of PV interneurons and decreased inhibitory activity in the prefrontal cortex [53]
4. Inflammatory cytokines can affect the synthesis of monoamine neurotransmitters; increase reuptake of dopamine, serotonin, and norepinephrine; and influence neurotransmitters' release [251]

Thus, the generation of prooxidants, on the one hand, is an element of the signaling pathways in the immune system. On the other hand, stimulation of the generation of ROS and H_2_O_2_ helps to maintain the immune response; however, in overexpression, it can lead to neuroinflammation. In combination with an insufficient antioxidant system, this leads to damage to the membranes of neurons and glia and a violation of their functions.

This is confirmed by the results of the study using a 31P-MRS-based method to directly quantify NAD+ and NADH concentrations in the brain at ultra-high-field MR scanners. This study confirmed a significant reduction in the NAD+/NADH ratio in chronically ill schizophrenia patients compared to a healthy control group and in first-episode schizophrenia patients compared to both a first-episode bipolar disorder patient group and a healthy control group. These findings provide evidence for redox imbalance in the brain in all phases of schizophrenia, potentially reflecting oxidative stress [278].

The development of oxidative stress induces the antioxidant system response. Activated microglia and macrophages release glutamate [274] in exchange for cysteine with an extracellular transporter (xc-transporter) [279]. During inflammation, activation of the xc-system functions as an endogenous antioxidant response since the influx of cysteine helps preserve the redox status of the cell [280], consequently increasing extracellular glutamate [35]. Several studies highlight the association between glutamate stimulation of microglia and decreased NOX activity by mGlu receptor activation [248].

Nevertheless, there is a decrease of canonical antioxidant systems, including peroxidases, oxidoreductases, oxidases, and dismutases, confirmed by multiple studies in schizophrenia [281, 282]. Recently, immunoglobulins with noncanonical properties have been discovered [283] that catalyze various reactions, including those similar to antioxidant enzymes. IgG antibodies with catalase-like activity in schizophrenia [284], IgG with peroxidase and oxidoreductase activities in healthy humans [285, 286], and IgG with SOD-like activity in patients with multiple sclerosis [287] were revealed. It can be assumed that such catalytic antibodies are involved in limiting oxidative damage at the inflammation areas, since antibodies can accumulate there. At the whole-organism level, catalytic antibodies with “antioxidant” properties can compensate for the deficiency of the canonical antioxidant systems in schizophrenia. Thus, human IgGs could probably also play a significant role in protecting humans from OS and toxic compounds.

### 4.8. ROS-Dependent Regulation of Neurotransmission and Changes Characteristic of Schizophrenia

Synaptic plasticity is characterized by the ability of the synapses to respond to stimulation and is determined by the change in the number of neurotransmitters or in the cell's ability to respond to neurotransmitters [288]. Controlled ROS production provides the optimal redox state for the activation of transductional pathways involved in synaptic changes. High ROS concentrations reportedly diminish synaptic signaling and brain plasticity mechanisms [289]. Intracellular calcium (Ca^2+^) is also one of the critical factors of synaptic plasticity in excitatory glutamatergic neurons [290].

Glutamate accounts for 50-60% of all neurotransmission in the brain, and the remaining 40-50% is GABAergic. Therefore, 90-99% of neurons are modulated by glutamatergic or GABAergic neurotransmission and less than 10% by other monoamine, neuropeptide, and neuroendocrine neurotransmissions. Most neural energy is expended on sustaining excitatory signaling within the CNS with action potential firing, and glutamatergic transmission is proposed to contribute as much as 80% of the total expenditure. There is evidence indicating an equally high need for cellular metabolism of inhibiting GABAergic interneurons [291]. Accardi et al. show that mitochondrial-derived reactive oxygen species (mROS) regulate postsynaptic GABAA receptors' strength at inhibitory synapses of cerebellar stellate cells. The generation of mROS has been traditionally linked to the cellular damage that accompanies the chronic disease. This data identifies mROS as a putative homeostatic signaling molecule, coupling cellular metabolism to the strength of inhibitory transmission [292]. As stated above, mitochondrial dysfunction plays a significant role in the pathogenesis of schizophrenia [293].

An increase in functional changes associated with the level of oxidative stress is shown in several neurotransmitter systems. Тhe site of redox modification on vulnerable molecules may undergo oxidative damage, resulting in an irreversible inhibitory action on proteins involved in synaptic transmission, as well as promoting mitochondrial dysfunction and excitotoxicity [294].

As mentioned above, glutamate is the major excitatory neurotransmitter of the central nervous system. Three groups represent ionotropic glutamate receptors, namely, AMPA, KA, and NMDA receptors, that mediate rapid transmembrane ion currents [295]. ROS and NO are essential mediators of NMDA receptor signaling [20]. Under normal physiological conditions, nitric oxide is generated by NMDA receptor-mediated activation of nNOS. In turn, nitric oxide regulates glutamate metabolism, the release of glutamate at the synapse, and transport out of the synapse [296]. Both NMDA receptor activation and blockade are reported to induce oxidative stress through an increase in NADPH oxidase (NOX) activity [297, 298]. Besides, the addition of glutamate to the culture of neurons at a concentration of 100 microns also causes the formation of ROS [299]. It has also been shown that prolonged exposure to glutamate damages motor neurons primarily through the activation of calcium/calmodulin, neuronal synthase, and nitric oxide in the cytoplasm and leads to the apoptosis of neurons [300]. In turn, increased ROS results in NMDA receptor hypofunction, and the production of nitric oxide can reduce NMDA receptor activity through S-nitrosylation of cysteines on NMDA receptor subunits. Similarly, S-nitrosylation of serine racemase inhibits the formation of the NMDA receptor coagonist, D-serine, which would decrease NMDA receptor activity [301].

In addition to NMDA receptors, *α*-amino-3-hydroxy-5-methyl-4-isoxazole propionic acid (AMPA) receptors represent the other dominant class of ionic glutamate receptors. It has been shown that the AMPA receptor's function is not directly related to the redox state, and lipid peroxidation does not affect its synaptic transmission [302]. In schizophrenia, changes in subunit expression of AMPA receptors and protein expression regulating the forward trafficking of AMPA receptors through the cell have been reported. These facts are the basis of the mechanism of impaired regulation of glutamate in schizophrenia. It has been revealed that in schizophrenia, abnormal trafficking of AMPA receptors from the endoplasmic reticulum to the synaptic membrane occurs [303, 304].

Glutamate signaling dysfunction and dysregulation of oxidative stress have been considered to play essential roles in schizophrenic prodrome [22]. The question of the primacy of oxidative stress in schizophrenia has not yet been definitively resolved. Still, most studies suggest that its influence is interdependent with the violation of glutamatergic neurotransmission [24, 305].

The effect of redox processes on GABAergic synaptic transmission and GABA release is very ambiguous. Redox state may have opposing effects on GABAB and GABAA receptors. For example, synaptic GABAA receptors can be weakened under oxidizing conditions; however, in the hippocampus, tonic currents are increased by hydrogen peroxide [306, 307]. Finally, oxidative damage can decrease the synthesis of GABA.

The hypofunction of subpopulations of GABAergic interneurons in the prefrontal cortex and hippocampus has long been established in schizophrenia. Also, there is a lot of data showing that redox changes of NMDA receptor synaptic input to inhibitory interneurons can decrease the release of GABA and induce the loss of inhibitory interneurons in the PFC (prefrontal cortex). And the modern view of the pathogenesis of schizophrenia is based on the hypofunction of the NMDA receptor and the loss of GABAergic neurons [308]. Recent experimental work carried out by the Department of Psychiatry at Harvard Medical School showed that interneurons in schizophrenia had a significantly smaller nucleus, which indicates an innate state of oxidative stress. The antioxidant N-acetylcysteine increased the area of the cell nucleus in interneurons in schizophrenia and eliminated synapse deficits [309].

It has long been known that the catabolism of dopamine shifts the cellular redox state toward oxidative stress through the production of superoxide, hydrogen peroxide, quinones, and quinoprotein adducts [310, 311]. In the presence of Mn, the autooxidation of dopamine produces semiquinones (SQ) and superoxide radicals (O_2_^-•^), as well as H_2_O_2_, which is readily converted to OH^•^ in the presence of Fe^2+^. On the other hand, the enzymatic oxidation of dopamine by monoamine oxidase (MAO) can also produce H_2_O_2_ and, subsequently, generate the toxic OH^•^ [35]. Dopamine metabolites, when interacting with Fe^3+^ and Cu^2+^, also produce ROS and thus exacerbate OS [311, 312].

It is now generally accepted that schizophrenia is based on a disruption of the interactions between the glutamatergic, GABAergic, and dopaminergic systems. At the present stage, an assumption is made that elevated baseline levels of dopamine observed in schizophrenia may be secondary to hypoglutamatergia. This is supported by evidence suggesting that NMDAR antagonists can enhance the release of dopamine and glutamate, leading to cortical disinhibition [313]. Besides, it was found that inhibition of NMDAR reduces the activity of putative GABA interneurons [314]. Thus, it is established that the main contribution to the pathogenesis of schizophrenia is made by mutually influencing disorders of neurotransmission of glutamate, dopamine, and GABA.

The contribution of other neurotransmitters to the pathogenesis of schizophrenia is less significant, but in any case, ROS signaling regulates neurotransmission to some extent. Noradrenaline in low concentrations, in contrast, increases neuroprotection for many types of neurons [315, 316]. There are isolated studies that indicate that OS disrupts the functioning of muscarinic cholinergic receptors, which is restored by GSH [317]. Acetylcholine, which is their mediator, is also involved in the development of schizophrenia [318]. Schizophrenia is also characterized by serotonin deficiency [319]. It has been shown that serotonin receptors can modulate dopaminergic functions, but their effect may have different tendencies. For example, serotonin can inhibit or stimulate the release of dopamine in the striatum [320, 321].

Thus, the entire neurotransmission of the CNS is regulated to some extent by ROS or RNS. All neurotransmitters are a close, mutually regulating system, and even with minor deviations from the norm, the homeostasis of this system is disrupted. Several morphological changes in cells in schizophrenia indicate that OS was present at an early stage of the morphosis. This area has already been studied in detail and is represented by a vast number of works. Here, we show only the most basic mechanisms. But the literature does not cover the following question: Are the described changes specific only for schizophrenia, or are they characteristic of other mental disorders?

### 4.9. Dysfunction of Parvalbumin-Positive Interneurons

Numerous studies, including postmortem, genetic, and in vivo electrophysiological experiments in murine models of schizophrenia, consider the disruption of parvalbumin- (PV-) positive GABAergic interneurons as a critical pathophysiological mechanism of schizophrenia [322]. PV interneurons are the most abundant type of GABAergic cells known for their high-frequency generation of action potentials [323]. PV interneurons form a common network that facilitates the processing of a massive flow of information from sensory systems and provides mechanisms for temporary memory, attention, learning, and social behavior. Parvalbumin is a Ca^2+^-binding protein that has little effect on excitatory potentials but significantly increases the decay rate of the signal [324]. This function allows maintaining a balance of excitatory/inhibitory postsynaptic potentials, synchronizing the activity of neurons. Electroencephalography- (EEG-) measured synchronized oscillatory activity, particularly in the gamma range, is abnormal in patients with schizophrenia [325].

Disorders of PV interneurons can lead to schizophrenia not only in adulthood but also at the stages of early neurogenesis. Recent studies have shown that suppressed PV interneuron activity decreases the survival and maturation of hippocampal neurons in newborns [326]. Caballero et al. showed that a slight suppression of PV interneuron activity in adolescence significantly reduces GABAergic transmission, disrupting the balance of excitation/inhibition processes in the prefrontal cortex [327]. An increase in the expression of PV interneurons in adolescence is necessary for better GABAergic regulation of excitation/inhibition, contributing to the activation of cognitive functions. Consequently, the loss of PV interneurons leads to an increase in the excitation/inhibition ratio due to the GABAergic interneurons' inability to maintain a high generation of action potentials, which leads to impaired processing of afferent information from the ventral hippocampus [328].

Correct high-frequency synchronization of PV interneurons requires a high level of metabolism and oxidative phosphorylation [329], which leads to an increase in ROS synthesis. PV interneurons are known to be extremely sensitive to increased ROS levels. In transgenic mouse models, it has been found that reduced glutathione (GSH) correlates with a deficiency of PV interneurons in the prefrontal cortex and hippocampus and disrupts neuronal synchronization [330]. Prefrontal cortical PV interneurons are more vulnerable to ROS during postnatal development. GSH deficiency results in a rapid and long-term decrease in the density of PV interneurons in the anterior cingulate gyrus [19]. Using an assessment of the level of oxidized and reduced glutathione in tissues, gamma fluctuations, and PV expression at different time points in transgenic Wistar rats, it was found that NMDA receptor (NMDAR) inhibition and GSH depletion during early postnatal development markedly alter gamma fluctuations [331]. It has been shown in vitro that during the early development of neurons, NMDAR inhibition primarily results in a sustained decrease in the peak gamma frequency. At the same time, a reduction of PV expression occurs only after a few days. These functional changes are expected to precede the suppression of PV expression. One of the possible mechanisms is assumed to be oxidative stress, which contributes to the loss of PV interneurons due to the suppression of internal antioxidant systems [332].

Damage to the perineuronal nets (PNNs) can be another critical factor in the disruption of PV interneurons. PNNs are reticular lattice structures composed of proteoglycans, chondroitin sulfate, hyaluronic acid, tenascin, and binding proteins, and braiding PV interneurons prevent the flow of cations spontaneously into the neuron [333]. The effect of oxidative stress on PNNs is to alter PV interneuron maturation and synapse formation [330]. Therefore, oxidative stress disrupts the plasticity of the PV interneuron network and may affect the ratio of excitatory and inhibitory synapses [334].

Mouse models were used to investigate the effects of oxidative stress on PV interneurons and PNNs. Indeed, mice with an alpha-7 nicotinic receptor (*α*-7-nAChR) deletion show reduced expression of PV, glutamate decarboxylase (GAD67), and NMDAR in PV interneurons [335]. *α*-7-nAChR activation inhibits NF-*κ*B-dependent pathways and mediates Nrf2-induced antioxidant responses, providing anti-inflammatory and neuroprotective effects [336]. Patients with schizophrenia have disturbances in neuregulin-1 (NRG1) and its receptor tyrosine kinase ErbB4 [337]. Signaling through the NRG1/ErbB4 complex controls the development of inhibitory chains in the cerebral cortex. Fazzari et al. have demonstrated that signaling through the ErbB4 receptor promotes the formation of perisomatic and axo-axonal synapses, and NRG1 mediates these effects [301]. ErbB4 is also required for the formation and maintenance of excitatory synapses on GABAergic interneurons. NRG1 suppresses NMDAR activation in the prefrontal cortex in subjects with schizophrenia, consistent with the increased NRG1-ErbB4 signaling observed in this disease [338]. NRG1 modulates NMDAR activity through tyrosine phosphorylation on the NR2 subunit. Enhanced NRG1 signaling may contribute to NMDAR hypofunction in schizophrenia [338].

Thus, the functioning of the PV interneuron, which plays an essential role in human cognitive functions impaired in schizophrenia, is directly dependent on the level of oxidative stress. As stated above, a decrease in the activity of the antioxidant system leads to inhibition of NMDAR and disrupts the PV interneurons, which makes a significant contribution to the pathogenesis of schizophrenia.

## 5. Antipsychotic Medication and Redox Correction in Schizophrenia

### 5.1. Antipsychotic Therapy Promotes Oxidative Imbalance

Antipsychotics are the drugs of choice for the long-term management of schizophrenia. As mentioned above, patients with schizophrenia have endogenous oxidative stress, which can cause the development of pathology. However, antipsychotic treatment can also cause oxidative imbalance.

Typical and atypical antipsychotics vary significantly in their effects on redox balance [339–342]. Animal studies indicate that typical antipsychotics most often cause oxidative stress. It was shown that treatment with haloperidol (45 and 90 days), a typical antipsychotic, significantly reduced the activity of Mn-SOD, Cu,Zn-SOD, and catalase and increased lipid peroxidation in the rat's brain [340]. But treatment with atypical antipsychotics (risperidone, clozapine, and olanzapine) did not cause any changes in antioxidant enzyme levels [340]. A 28-day study in male Wistar rats that received haloperidol daily showed an increase in thiobarbituric acid (TBA) reactive substances (TBAR) inducing the production of superoxide in the hippocampus [343]. In other experiments in rats after the administration of haloperidol, an increase in the level of TBAR in the striatum, but a decrease in the cortex, was also observed [341, 342]. The long-term treatment (90 and 180 days) with haloperidol also significantly decreased the levels of Mn-SOD, Cu,Zn-SOD, and to a lesser extent, catalase [339]. In the same work, it was shown that atypical antipsychotics such as ziprasidone, risperidone, and olanzapine did not show significant changes in the lipid peroxidation products (hydroxyalkanals) after 90 days of treatment [339]. However, further treatment (up to 180 days) resulted in significantly increased levels of hydroxyalkanals in ziprasidone and risperidone, but not in olanzapine-treated rats [339]. These data indicate that long-term treatment, even with atypical antipsychotics, can contribute to prooxidant processes. At the same time, posttreatment with some atypical antipsychotics for 90 days after 90 days of typical antipsychotic treatment restores developed redox abnormalities [339]. These observations can be explained by the direct antioxidant activity of some atypical antipsychotics, such as olanzapine and clozapine [344].

Summarizing the available data from animal studies, we can conclude that oxidative damage of the brain during treatment with typical antipsychotics is due to the following mechanisms:

- A significant decrease in the activity of Mn-SOD, Cu,Zn-SOD, and catalase
- Increased lipid peroxidation of membranes
- Reduction of NO concentration [345]
- Increased production of superoxide and H_2_O_2_ [345]

Human studies also indicate that antipsychotics contribute to oxidative stress [346]. It has been shown that MDA levels in patients treated with risperidone, amisulpride, quetiapine, and clozapine were significantly lower than in the first-generation antipsychotic group [347]. Treatment with typical antipsychotic haloperidol was associated with higher serum TBAR and lower antioxidant parameters in patients with schizophrenia [348]. Besides, an overdose of typical antipsychotics can cause oxidative stress [349]. Another study reported that both typical and atypical antipsychotics contributed to patients' prooxidant state with various types of schizophrenia [350]. The specific activity of SOD and glutathione peroxidase was reduced, but MDA levels were increased in chronic patients, regardless of the type of antipsychotic [350]. Padurariu et al. [351] obtained similar results. An increase in the specific activity of SOD, mainly in patients receiving haloperidol and quetiapine, was shown [351]. Antipsychotic treatment also affects peripheral nonenzymatic antioxidants. Indeed, uric acid levels did not change after treatment, while albumin and total bilirubin levels decreased significantly after treatment [352]. Thioredoxin levels also decreased in patients receiving antipsychotic medication [353]. However, no correlation was found between thioredoxin levels and treatment with atypical antipsychotics [353]. Antipsychotic treatment also affects the serum lipid profile [354]. Indeed, serum lipid profiles of patients with first-episode psychosis before and after seven months of treatment were significantly different [354]. Our data also confirm the effect of therapy on the activity of antioxidant systems in schizophrenia [355]. We showed that atypical antipsychotics do not have a pronounced impact on the glutathione system, while treatment with typical antipsychotics leads to a further decrease in reduced glutathione, thereby exacerbating the imbalance in metabolic processes in schizophrenia [355]. Besides, metabolic syndrome and antipsychotic-induced weight gain may be associated with high levels of oxidative stress in patients [356].

However, there is clinical evidence of a decrease in oxidative stress markers after the antipsychotic treatment of schizophrenia patients [357–359]. Therapy with atypical antipsychotics improved the redox balance, together with the results of the Brief Psychiatric Rating Scale for schizophrenia [359]. The antipsychotic medication (7 months) of first-episode psychosis patients not only had a significant anti-inflammatory effect but also reduced lipid peroxidation and protein oxidation-related indices of oxidative stress [357]. A decrease in the neopterin levels and an increase in antioxidant levels were found after three months of treatment with antipsychotics [358]. Zhang et al. published evidence that clozapine treatment significantly increased SOD and decreased lipid peroxidation in patients with schizophrenia [350]. In our work, it was shown that therapy with typical antipsychotics leads to normalization of catalase activity. However, the activity of glucose-6-phosphate dehydrogenase continues to decrease [360]. Besides, we showed that antipsychotic therapy leads to a decrease in MDA in red blood cells and blood plasma but does not affect the level of oxidized and reduced glutathione [361].

Thus, despite contradictory data, nevertheless, there is an association between antipsychotic therapy and oxidative stress. Both typical and atypical antipsychotics have the most significant effect on redox balance in schizophrenia. Therefore, additional antioxidant treatment can have beneficial effects on the redox balance and, accordingly, on the condition of schizophrenia patients.

### 5.2. Antioxidant Therapeutics in Schizophrenia

Antioxidant treatment can be used at various stages of schizophrenia, from prenatal and postnatal development to acute and chronic phases of the illness [362]. However, the most realistic option is to use antioxidant therapy to treat the disease's active stage.

To assess the promise of prescribing antioxidants to patients with schizophrenia in the last decades, many different studies have been conducted (Table 2). Some effects of antioxidants were analyzed in detail in large-scale meta-analyses [36, 363, 364]. It was shown that additional antioxidant therapy could improve the mental state of patients with schizophrenia.

N-Acetylcysteine (NAC) is an L-cysteine precursor, an antioxidant, and a free radical-scavenging agent. NAC can regulate glutathione (GSH): this is a precursor for glutathione synthesis and a stimulator of the cytosolic enzymes involved in glutathione regeneration [365]. NAC is also able to regulate excessive brain glutamate through the cysteine-glutamate antiporter [366]. Symptoms of schizophrenia are associated with elevations in glutamatergic metabolites across several brain regions [367] and oxidative stress [40]. This determines the potential possibility of the use of NAC as adjunctive therapy for patients with schizophrenia [368–377]. The NAC study's frequent outcome as an adjunct to antipsychotic treatment was a significant reduction in PANSS total and the negative PANSS subscale, but not positive symptoms or cognition (Table 2). Also, some authors noted an improvement in the generation of negative mismatch (MMN) and working memory performance in patients with schizophrenia (Table 2). A recent meta-analysis of randomized controlled trials with N-acetylcysteine in the treatment of schizophrenia confirmed the effectiveness of NAC, but it is worth noting that therapeutic effects were observed at a later point in time (>24 weeks) [364]. This suggests that long-term administration of N-acetylcysteine is required for successful treatment.

Ginkgo biloba is the most widely studied plant antioxidant in the treatment of schizophrenia [378–381]. Published studies and meta-analyses demonstrated that Ginkgo as an adjunctive therapy could alleviate the symptoms of chronic schizophrenia and improve tardive dyskinesia (Table 2).

Selegiline and allopurinol have also been studied as an additional antioxidant treatment for schizophrenia patients [382–388]. In selegiline, the ability to reduce negative symptoms was noted; on the contrary, in allopurinol, the ability to interfere with positive symptoms was noted. However, the use of these drugs often did not make a performance compared with the control group (Table 2).

Vitamin E is a lipid-soluble antioxidant. Evaluation of the effectiveness of treatment with vitamin E in patients with schizophrenia showed a significant difference in Abnormal Involuntary Movement Scale (AIMS) score (Table 2) [389–395]. There is also evidence that vitamin E is more effective for patients using classic antipsychotics compared to patients using atypical antipsychotics [36].

There is very little data on the individual use of vitamin C as an antioxidant therapy for schizophrenia [396]. But since ascorbic acid has a synergistic effect with vitamin E, there is evidence of the effect of their combined action in patients with schizophrenia [397]. The effects are similar to those of vitamin E. Supplements with polyunsaturated fatty acids (PUFAs) in schizophrenia help to reduce the PANSS scores [398–403], but in some cases [404, 405] the changes are not significant (Table 2). Despite all efforts, PUFA mechanisms of action are still poorly understood.

### 5.3. Promising Transcription Factor-Targeting Drugs

Insufficient efficiency of antioxidant therapy stimulates the search for new approaches to correct redox balance. The potential new therapeutic avenues are associated with activators of redox-regulated transcription factors (Nrf2, FoxO). The activation of Nrf2 or FoxO by low-molecular-weight drugs may have therapeutic potential to control redox balance by enhancing endogenous antioxidant responses. Numerous drugs such as dimethyl fumarate (DMF), sulforaphane, cyanoenone triterpenoids (in particular, bardoxolone methyl and omaveloxolone), nitro fatty acids, and hydroxylamine are considered promising Nrf2 activators [417]. Of these, only DMF was approved by the US Food and Drug Administration and the European Medicines Agency to treat remitting-relapsing MS. [417] Sulforaphane, curcumin, resveratrol, and metformin have been tested to treat schizophrenia (Table 2). The Keap1 inhibitor sulforaphane increased blood and brain GSH levels in healthy humans [406] and improved cognitive impairments in schizophrenia individuals [407]. Sulforaphane exhibited an atypical antipsychotic activity in an animal model [408]. Another Keap1 inhibitor, curcumin, reduced total PANSS and the negative symptom subscale scores in schizophrenia [410]. Another Nrf2 activator (DDO-7263) showed a neuroprotective and anti-inflammatory effect through Nrf2 activation and NLRP3 inflammasome inhibition in an animal model [409]. It is noteworthy that there are some reversible Keap1-binding compounds with low off-target activity that protect cells from oxidative effects by preserving the ATP content and mitochondrial potential in the cell culture of primary astrocytes [418].

Some drugs can modulate the FoxO transcription factor activity (Table 2). Resveratrol activates NAD-dependent deacetylase sirtuin-1 (SIRT1), and thereby activates the transcription factor FoxO, and also inhibits NF-*κ*B. Resveratrol demonstrated anxiolytic and antipsychotic potentials in murine models of anxiety and schizophrenia [413]. However, oral resveratrol is not effective in human trials [411, 412]. Another promising compound with a similar mechanism of action is salvianolic acid B, which has been shown to alleviate depression-like symptoms and cognitive deficits in animal models [154, 414, 415]. Metformin, an antidiabetic agent, was identified as a therapeutic activator of FoxO3. Metformin showed efficacy in the treatment of antipsychotic-induced weight gain, dyslipidemia, and metabolic abnormalities in schizophrenia [416]. Besides, there are specific FoxO activators. Cautain et al., using image-based high-content screening technology, identified the isothiazolonaphthoquinone-based compound (LOM612) as a specific FoxO3a protein activator [419]. This compound induced nuclear translocation of FoxO3a and FoxO1 proteins and also did not affect the translocation of NF-*κ*B in U2OS cancer cells [419]. Some inhibitors of PI3K, Akt, and other protein kinases may also be considered as possible regulators of FoxO activity [420].

### 5.4. Stratification of Patients Based on Oxidative Stress-Related Markers for the Administration of Antioxidant Treatment

Heterogeneity and complexity are the main obstacles to developing etiopathogenetic treatments for schizophrenia. The lack of effectiveness of traditional antipsychotic therapy, especially in the treatment of negative and cognitive symptoms, leads to the search for new therapeutic avenues. Considering the above data about the involvement of oxidative stress in the molecular mechanisms of the schizophrenia pathogenesis, redox correction in conjunction with antipsychotic therapy is a promising therapeutic strategy. However, existing treatment approaches do not consider the multistage process of schizophrenia. Indeed, the redox markers in first-episode psychosis patients, people with acute relapse of psychosis, chronic inpatients, and stable medicated outpatients, as well as in people with different types of schizophrenia and depending on antipsychotic treatment, are different [40, 350]; therefore, it is necessary to take into consideration the characteristics of each person's redox status, including during clinical trials.

As stated above, clinical trial results indicate that additional antioxidant therapy has beneficial effects, including for treatment-resistant schizophrenia patients [421]. However, a shift in the redox balance towards an increase in the reduction potential leads to reductive stress and a paradoxical increase in ROS production [422]. Therefore, the appointment of antioxidant therapy should be strictly controlled. It is also necessary to consider that prolonged use of antioxidant drugs can have prooxidant effects [422]. Consequently, stratification criteria based on oxidative stress-related markers need to be developed. The stratification criteria may be blood-based biomarkers, since they may reflect the degree of redox imbalance in the brain, or noninvasive visualization-based markers. Genetic-based stratification may also be promising. Identification of subgroups of high-risk patients with common redox imbalance parameters will allow choosing the optimal targeted treatment strategy for each patient. This approach is an important step towards personalized and precision medicine.

## 6. Conclusions

The heterogeneity of schizophrenia is reflected in the diversity of oxidative stress-related mechanisms that contribute to the disease. In our opinion, genetic causes lead to a predisposition to redox imbalance. There is ample evidence that a variety of environmental factors contribute to the dysregulation of gene expression caused by abnormal regulation of redox-sensitive transcriptional factors, noncoding RNAs, and epigenetic mechanisms. These changes contribute to altered redox signaling. Thus, these processes form the basis of the redox imbalance and lead to mitochondrial dysfunction and metabolic abnormalities that contribute to aberrant neuronal development, abnormal myelination, NMDA receptor hypofunction, and dysfunction of parvalbumin-positive interneurons. Immune dysregulation through various mechanisms also enhances redox imbalance. All these mechanisms ultimately contribute to the phenotypic realization of predisposition to redox imbalance and the manifestation of schizophrenia. However, it should be noted that all these mechanisms are interconnected and, at the same time, can act both independently and jointly in different periods. But these mechanisms eventually increase the likelihood of developing schizophrenia. Knowledge of oxidative stress-related mechanisms could pave the way for novel treatment options in schizophrenia. We suggest that not only classical antioxidants but also transcription factor-targeting drugs have great promise in schizophrenia. Since dysregulation of redox-sensitive transcriptional factors (e.g., Nrf2, NF-*κ*B, and FoxO) may play an important role in the development of schizophrenia, modulators of the activity of these factors may contribute to the normalization of redox balance. However, it is necessary to use redox regulatory drugs with caution, since any change in the redox balance towards oxidative stress and reductive stress has detrimental consequences. Therefore, we propose to develop the stratification criteria of schizophrenic patients based on oxidative stress-related markers for the administration of antioxidant treatment for high-risk patients. Nevertheless, there are still many unresolved questions about the role of oxidative stress in schizophrenia pathogenesis. We expect that further research will reveal new oxidative stress-related mechanisms in schizophrenia.

## Figures and Tables

**Table 1:** Changes in oxidative stress-related markers in schizophrenia.

Parameters	Reviews			Meta-analyses				
	Yao J. K. and M. S. Keshavan, 2011 [35]	Boskovic M. et al., 2011 [36]	Koga M. et al., 2016 [37]	Zhang M. et al., 2010 [38]	Flatow J. et al., 2013 [40]	Fraguas D. et al., 2017 [43]	Fraguas D. et al., 2019 [45]	Carvalho A. F. et al., 2020 [46]
*Nonenzymatic antioxidant system*								
Bilirubin	↓		↓					
Biopyrrins*^α^*	↑		↓↑					
Thioredoxin	↑		↓↑					
Uric acid	↓	↓	↓		↓*^ν^* ↓*^σ^*		N.S.	
Ascorbic acid (vitamin C)*^α^*	↓	↓	↓		↓*^σ^*			
Tocopherol (vitamin E)*^α^*	↓	↓	↓		↓*^σ^*			
Pyridoxal (vitamin B6)								↓
Folate (vitamin B9)								↓
Glutathione*^β^*	↓		↓			N.S.	N.S.	
Free thiols	↓		↓					
PUFAs*^γ^*	↓		↓				↓*^ν^*	↓
*Enzymatic antioxidant system*								
Superoxide dismutase*^δ^*	↓↑	↓↑	↓↑	↓	↓*^μ^* ↓*^ν^* ↓*^π^* ↓*^σ^*	N.S.	N.S.	
Catalase*^δ^*	↓	↓	↓↑	N.S.	↓*^μ^* ↓*^ν^* ↑*^π^* ↓*^σ^*	N.S.	N.S.	
Glutathione peroxidase*^δ^*	↓↑	↓	↓↑	N.S.	↓*^μ^* N.S.*^ν^* N.S.*^π^* ↓*^σ^*	N.S.	N.S.	
Glutathione reductase*^δ^*		↓	↑					
Glutathione transferase*^δ^*			↑					
*Free radical oxidation product markers*								
Thiobarbituric acid reactive substances (TBARS)*^ε^*	↑	↑	↑	↑	↑*^ν^* ↑*^π^* ↑*^σ^*		N.S.	↑
Lipid peroxides	↑							
Pentane*^θ^*	↑		↑					
Ethane*^θ^*	↑		↑					
Isoprostanes*^θ^*	↑							
Carbonyl groups		↑	↑					
4-Hydroxynonenal	↑		↑					
3-Nitrotyrosine	↑	↑	↑					
8-Hydroxy-2-deoxyguanosine*^λ^*		↑						
*Other markers*								
NO	↑	↑		↑	↓*^ν^* ↑*^π^*			
Nitric oxide synthase*^δ^*	↓↑		↓↑					
Homocysteine		↑					↑*^ν^*	
Xanthine oxidase*^δ^*		↑						
Total antioxidant capacity			↓					
The ferric reducing ability of plasma			↓					
Total antioxidant potential			↓					
Total oxidant status		N.S.						
Total antioxidant status	↓		↓		↓*^ν^*	N.S.	↓*^ν^*	

*Notes*. Data are for plasma/serum unless otherwise indicated. *^α^*In plasma/serum or urine. *^β^*In plasma/serum, red blood cells, or brain tissues. *^γ^*In red blood cell membrane. *^δ^*In plasma/serum, red blood cells, platelets, or postmortem brain. *^ε^*In blood, plasma, cerebrospinal fluid, or red blood cells. *^θ^*In exhaled air. *^λ^*In urine or postmortem brain. *^μ^*Acute relapse of psychosis. *^ν^*Drug-naïve first-episode psychosis. *^π^*Stable medicated outpatients. *^σ^*Chronic inpatients. Abbreviations: PUFAs = polyunsaturated fatty acids. NO = nitric oxide. N.S. = not significant.

**Table 2:** The results of the use of redox regulatory drugs in schizophrenia and animal models.

Drugs	Сlass	Mechanism of action	Studies	Outcome
*Antioxidant drugs*				
N-Acetylcysteine (NAC)	Cysteine precursor	NAC (i) Activates the synthesis of glutathione, the main endogenous antioxidant mediator in the brain (the most important mechanism) (ii) Has its own nonspecific activity in neutralizing various free radical groups	Yolland et al., 2020 (meta-analysis) [364]	Total PANSS and in the negative PANSS subscale and total scores as well as the cognitive domain of working memory were significantly improved with N-acetylcysteine supplementation after 24 weeks of treatment
			Berk et al., 2008 [368]; Farokhnia et al., 2013 [369]	Significant reduction of the Positive and Negative Syndrome Scale (PANSS) negative, PANSS total, and Clinical Global Impression (CGI) scales in comparison to placebo
				No significant change on the PANSS positive subscale was seen
				There was no significant difference with the control group in the frequency of side effects.
			Lavoie et al., 2008 [370]	Administration of NAC to schizophrenia patients resulted in improved auditory cortical functioning, improved mismatch negativity (MMN) generation
			Zheng et al., 2018 [371]	Adjunctive NAC significantly improved total (positive and negative) symptoms in schizophrenia
			Rapado-Castro et al., 2017 [372]	Significantly higher working memory performance compared with placebo
			Sepehrmanesh et al., 2018 [373]	Significant improvement in total PANSS and in the positive and negative PANSS subscales
				Improvements in attention, short-term and working memory, executive functioning and speed of processing
			Conus et al., 2018 [374]	NAC therapy improved neurocognition and reduced positive symptoms among patients with high peripheral oxidative status
				No changes in negative or positive symptoms or functional outcome were observed with NAC
			Klauser et al., 2018 [375]	Significant improvements in neurocognition and a reduction of positive symptoms
				Increase in GSH levels in the medial prefrontal cortex
			Breier et al., 2018 [377]	Significant improvement in PANSS total, negative, and disorganized thought symptom scores
				No changes in PANSS positive symptoms and BACS cognitive scores
			McQueen et al., 2020 [376]	A single dose of NAC was associated with decreases in rs-FC in prefrontal cortical regions of the DMN and SN network in patients with established schizophrenia
Ginkgo biloba extract	Contains flavonoids, tricyclic diterpenes (ginkgolides A, B, C, and J), sesquiterpenes (bilobalide A)	Ginko biloba extract (i) Scavenger of superoxide anion, hydroxyl radicals, peroxyl (ii) Reduces the concentration of primary and secondary products of lipid peroxidation (diene conjugates, TBA-active products) (iii) Induces the enzymatic activity of superoxide dismutase and catalase	Singh et al., 2010 (meta-analysis) [381]	Statistically significant moderate improvement in total and negative symptoms of chronic schizophrenia
			Doruk et al., 2008 [378]	Significant reduction of the PANSS negative
				No significant change on the PANSS total and in the positive PANSS subscale
			Zhang et al., 2011 [379]	A significant decrease in the Abnormal Involuntary Movement Scale (AIMS) total score
				No between-group differences in the PANSS total score or cognitive measures from baseline
			Rathbone et al., 2005 [380]	Treatment with Ginkgo biloba resulted in moderate improvement in total and negative symptoms of schizophrenia
Selegiline	Selective inhibitor of MAO-B	The antioxidant effect of selegiline may be associated with the protection of neurons from oxygen free radicals that are released as a result of MAO-B activity	Amiri et al., 2008 [382]	Decline of negative symptoms and PANSS total scores
			Bordbar et al., 2008 [383]	No significant change on the PANSS negative subscale was seen
			Bodkin et al., 2005 [384]	Significant improvements in negative symptoms
Allopurinol	Xanthine oxidase inhibitor	Allopurinol prevents the formation of free radicals: (i) Inhibits the xanthine oxidase reaction, during which superoxide anion radical is actively formed	Akhondzadeh et al., 2005 [385]	A significant superiority in the treatment of positive symptoms, general psychopathology symptoms as well as PANSS total scores
				Decreased Extrapyramidal Symptoms Rating Scale (ESRS)
			Brunstein et al., 2005 [386]	Significant improvement in PANSS total, positive, negative, and general scores, particularly for positive symptoms
			Dickerson et al., 2009 [387]	At least a 20% reduction in total PANSS score
			Weiser et al., 2012 [388]	No differences with control group on the PANSS
Vitamin E	Vitamin	Vitamin E is a natural antioxidant that neutralizes free radicals: (i) There is a transfer of hydrogen of the phenyl group to the peroxide radical (ii) Performs structural function by interacting with phospholipids of biological membranes	Adler et al., 1993 [389]	Significant reduction of AIMS score
			Adler et al., 1999 [390]	No significant reduction of AIMS and Brief Psychiatric Rating Scale (BPRS)
			Dorevitch et al., 1997 [391]	No significant differences between vitamin E and placebo-treated patients in AIMS score
			Lohr et al., 1996 [392]	Significant reduction of AIMS score
				Significant change on the PANSS positive subscale
			Zhang et al., 2004 [393]	Significant reduction of AIMS score
			Sajjad, 1998 [394]	Significant reduction of AIMS score
			Soares-Weiser et al., 2018 [395]	No clear difference between vitamin E and placebo for the outcome of TD (not improved to a clinically important extent)
Vitamin C	Vitamin	Vitamin C antioxidant activity: (i) The formation of a redox pair of ascorbic acid/dehydroascorbic acid, restores the active form of vitamin E (ii) Prevents or reverses the oxidation process of reduced glutathione (GSH) to its functionally inactive form (GSSG)	Dakhale et al., 2005 [396]	Significant change on the BPRS
Vitamins C and E	Vitamins	Ascorbic acid has a synergistic effect for vitamin E: (i) Restores the oxidation product of tocopherol (*α*-tocopheroxide) to *α*-tocopherol	Nicolaus et al., 2002 [397]	Significant reduction in dyskinetic movement total score High dietary intake will lead to prooxidant action
Vitamins E and C and fatty acids	Vitamins and dietary supplements	Q.V. mechanisms of action of vitamins E and C and fatty acids	Arvindakshan et al., 2003 [398]	Significant reduction of PANSS and BPRS and increase of QOL (Henrich's Quality of Life scale)
			Sivrioglu et al., 2007 [399]	Significant reduction of BPRS, Scale for Assessment of Negative Symptoms, Simpson-Angus Rating Scale, and Barnes Akathisia Rating Scale
Polyunsaturated fatty acids (PUFAs)	Dietary supplements	The detailed mechanisms of action are mostly unknown, but PUFAs have anti-inflammatory and antiatherogenic effects	Emsley et al., 2002 [400]	Significant reduction in PANSS total scale
			Emsley et al., 2006 [404]	No significant difference in the Extrapyramidal Symptom Rating Scale
			Fenton et al., 2001 [405]	No significant change in PANSS, Abnormal Involuntary Movement Syndrome scale, Clinical Global Impression scale
			Peet et al., 2001 [401]; Peet et al., 2002 [402]	Significant reduction in PANSS total
			Amminger et al. 2010 [403]	Significantly reduced positive, negative, and general symptoms and improved functioning compared with placebo

*Transcription factor-targeting drugs*				
Sulforaphane	Nrf2 transcription factor activator	Inhibit Keap1 protein through electrophilic modification, which leads to Nrf2 activation. The transcription factor Nrf2 plays a central role in the inducible expressions of many cytoprotective genes in response to oxidative stress	Sedlak et al. 2017 [406]	Isothiocyanate sulforaphane increased blood and brain GSH levels in healthy human subjects following 7 days of administration
			Shiina et al. 2015 [407]	Sulforaphane may have the potential to improve cognitive impairments in patients with schizophrenia, but the differences before and after treatment are not significant
			Shirai et al., 2015 (animal model) [408]	Sulforaphane exhibited an atypical antipsychotic activity in PCP-induced cognitive deficits in animal models. Genetic analysis showed an epistatic interaction between Nrf2 and Keap1 gene variants on working memory in schizophrenia
DDO-7263	Nrf2 transcription factor activator	Nrf2 activation and NLRP3 inflammasome inhibition	Xu et al., 2019 (animal model) [409]	The neuroprotective effects of DDO-7263 have been proven in mice, through Nrf2 activation and NLRP3 inflammasome inhibition
Curcumin	Polyphenolic compound	Inhibit Keap1 protein through electrophilic modification, which leads to Nrf2 activation	Miodownik et al. 2019 [410]	Significant reduction in total PANSS and in the negative symptoms subscale. There were no differences in the positive and general PANSS subscales, and the Calgary Depression Scale for Schizophrenia scores
Resveratrol	Anthocyanins	Resveratrol is (i) An activator of NAD-dependent deacetylase sirtuin-1 (SIRT1), which activates the transcription factor FoxO (ii) An inhibitor of NF-*κ*B (anti-inflammatory effect)	Zortea et al., 2016 [411]	Oral resveratrol in sufficiently low doses has not brought improvement
			Zortea et al., 2016 [412]	No significant improvement in psychopathology severity
			Magaji et al., 2017 (animal model) [413]	Anxiolytic and antipsychotic potentials of resveratrol in murine models of anxiety and schizophrenia in mice was found
Salvianolic acid B	Polyphenolic compound	Salvianolic acid B like resveratrol is an activator of SIRT1 and an inhibitor of NF-*κ*B	Huang et al., 2019 (animal model) [414]	Study has confirmed the antidepressant activity in an induced depression rat model
			Yu et al., 2016 (animal model) [415]	Treatment of stress-challenged rats with fluoxetine and fluoxetine combined with salvianolic acid could alleviate depression-like symptoms and cognitive deficit
			Liao et al., 2020 (animal model) [154]	SalB relieved CMS-induced depressive-like state in mice through the mitigation of inflammatory status, oxidative stress, and the activation of the AMPK/SIRT1 signaling pathway
Metformin	Antidiabetic agent	Activate FoxO3 via AMP-activated protein kinase activation	Jiang et al., 2020 [416]	Significantly reduce antipsychotic-induced weight gain, dyslipidemia, and metabolic abnormalities in schizophrenia

## Data Availability

The presented data supporting this systematic review are from previously reported studies and datasets, which have been cited. The processed data are available from the corresponding author upon request.
